# Synthesis of Some Novel Thiadiazole Derivative Compounds and Screening Their Antidepressant-Like Activities

**DOI:** 10.3390/molecules23040716

**Published:** 2018-03-15

**Authors:** Nafiz Öncü Can, Özgür Devrim Can, Derya Osmaniye, Ümide Demir Özkay

**Affiliations:** 1 Department of Pharmaceutical Analytical Chemistry, Faculty of Pharmacy, Anadolu University, 26470 Eskişehir, Turkey; nafizoc@anadolu.edu.tr; 2 Doping and Narcotic Compounds Analysis Laboratory, Faculty of Pharmacy, Anadolu University, 26470 Eskişehir, Turkey; dosmaniye@anadolu.edu.tr; 3 Department of Pharmacology, Faculty of Pharmacy, Anadolu University, 26470 Eskişehir, Turkey; udemir@anadolu.edu.tr; 4 Department of Pharmaceutical Chemistry, Faculty of Pharmacy, Anadolu University, 26470 Eskişehir, Turkey

**Keywords:** activity cage, modified forced swimming, tail-suspension, thiadiazole

## Abstract

Novel thiadiazole derivatives were synthesized through the reaction of acetylated 2-aminothiadiazole and piperazine derivatives. The chemical structures of the compounds were clarified by Infrared Spectroscopy (IR), ^1^H Nuclear Magnetic Resonance Spectroscopy (^1^H-NMR), ^13^C Nuclear Magnetic Resonance Spectroscopy (^13^C-NMR) and Electronspray Ionisation Mass Spectroscopy (ESI-MS) spectroscopic methods. Antidepressant-like activities were evaluated by the tail-suspension (TST) and modified forced swimming (MFST) methods. Besides, possible influence of the test compounds on motor activities of the animals were examined by activity cage tests. In the TST, administration of the compounds **2c**, **2d**, **2e**, **2f**, **2g** and **2h** significantly decreased the immobility time of mice regarding the control values. Further, in the MFST, the same compounds reduced the total number of immobility behaviors while increasing swimming performance. However, no change was observed in the total number of climbing behaviors. These data suggested that compounds **2c**, **2d**, **2e**, **2f**, **2g** and **2h** possess notable antidepressant-like activities. Reference drug fluoxetine (10 mg/kg) was also exhibited its antidepressant activity, as expected. No significant difference was seen between the locomotor activity values of the test groups signifying that observed antidepressant-like activities are specific. Theoretical calculation of absorption, distribution, metabolism, excretion (ADME) properties for the obtained compounds were performed and obtained data supported the antidepressant-like potential of these novel thiadiazole derivatives.

## 1. Introduction

Word Health Organization (WHO) describes depression as a “mental disease characterized by depressed mood, loss of pleasure/interest, low self-worth, feel of guilty, reduced energy, poor concentration, disturbed sleep and appetite.” Women gender, genetic predisposition, economic disadvantages, social disadvantages (e.g., low education), destructive life experiences (e.g., exposure to violence, being separated or divorced) and chronic illness are among the main risk factors for this disorder [1].

It is well known that incidences of depression and other emotional disorders increases worldwide. Estimation of WHO indicates that depression have an increasing portion in the worldwide burden of disease, rising from third place in 2004 (4.3% of the total) to first place by 2030 (6.2% of the total). Lifetime prevalence rates of this disease in most countries are estimated in the range of 8 to 12 percent. Significant socioeconomic costs of depressive disorders are also underlined in the reports of WHO [2].

As summarized in the WHO mhGAP Intervention Guide, prevailing strategies for the management of depression involve basic psychosocial support together with antidepressant treatment or psychotherapy (e.g., interpersonal psychotherapy, cognitive behavioral therapy etc.) [1]. Selective serotonin reuptake inhibitors (such as paroxetine, fluoxetine, fluvoxamine, sertraline, citalopram), serotonin modulators and stimulators (vilazodone, vortioxetin), serotonin antagonists and reuptake inhibitors (trazodone), serotonin/noradrenaline reuptake inhibitors (milnacipran, duloxetine, venlafaxine etc.), noradrenaline reuptake inhibitors (viloxazine, reboxetine), tricyclic antidepressants (nortriptyline, amitriptyline, protriptyline clomipramine, imipramine, desipramine etc.), tetracyclic antidepressants (amoxapine, maprotiline, mianserin, mirtazapine etc.) and monoamine oxidase inhibitors (phenelzine, tranylcypromine, moclobemide etc.) are the main classes of currently prescribed antidepressant drugs. On the other hand, these conventional antidepressants have some disadvantages, such as delayed onset of therapeutic effect, high ratio of non-responding patients and undesirable side effects [3]. Therefore, discovering and developing new antidepressant drugs are of great importance for providing new choices for pharmacotherapy of depression and related psychiatric disorders.

Based on this fact, our research group targeted to design some new molecules in order to develop potent and safe antidepressant drug candidates. We preferred to derivate thiadiazole ring for obtaining novel molecules because, derivatives of this 5-membered di-unsaturated ring are known to possess significant pharmacological effects on central nervous system. For instance, centrally mediated antinociceptive [4,5,6,7], anticonvulsant [8,9,10,11], anxiolytic [10,11,12] and antidepressant-like [4,10,11,12,13] activities of thiadiazole derivative compounds have been demonstrated, so far. Therefore, based on the pharmacological efficacy potential of thiadiazole derivative compounds on emotion and behavior, we synthesized some novel molecules and screened their antidepressant effects by using some validated in vivo tests.

## 2. Results and Discussion

**Figure 1:**
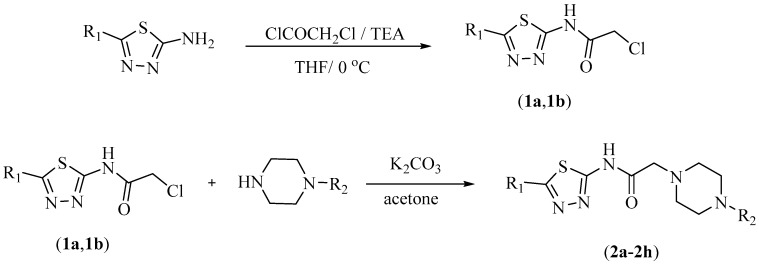
Synthesis pathway of target compounds.

Synthesis procedure to obtained compounds **2a**–**2h** were performed as summarized in Figure 1. Firstly, 2-chloro-*N*-(5-methyl/ethyl-1,3,4-thiadiazol-2-yl)acetamide (**1a**, **1b**) were prepared with acetylation of 5-methyl/ethyl-1,3,4-thiadiazol-2-amine. Secondly, reaction of compounds **1a, 1b** and an appropriate piperazine derivatives afforded target compounds (**2a**–**2h**). In the IR spectra, stretching absorptions belonging to amide bond was determined at 3282–3373 cm^−1^. The stretching absorption at about 1699–1707 cm^−1^ were registered proved C=O bond of the amide group. ^1^H-NMR spectra, aromatic protons of benzene (**2d**, **2h**) were observed between 6.77 and 7.21 ppm. Methyl protons of thiadiazole in compounds **2a**, **2b**, **2c**, **2d** were recorded between 2.60 and 2.61 ppm as a singlet. Ethyl protons of thiadiazole in compounds **2e**, **2f**, **2g**, **2h** were recorded between 1.27 and 1.33 ppm as a triplet and between 3.01 and 3.20 ppm as a quartet. In the ^13^C-NMR, carbonyl carbon gave a peak between 168.93 and 174.81 ppm. When the aliphatic region is examined, the aliphatic carbons of the substituents are observed in the range of 12.46–65.25 ppm. In the mass spectrum, all masses were accorded with the estimated M + H values. The IR, ^1^H-NMR, ^13^C-NMR, and MS spectra of all compounds are presented in supplementary file (Spectra 1–32).

Evaluate pharmacokinetic properties of drug nominees is important drug discovery approach during the early drug discovery phases. Recently, number of drug candidates with clarified absorption, distribution, metabolism, excretion (ADME) properties, has significantly increased by the help of combinatorial chemistry [14].

In the present study, the online Molinspiration chemical property software was used in an attempt to calculate ADME properties of the compounds **2a**–**2h** [15]. This program is based upon to principle of Lipinski’s rule of five. Lipinski’s five-rule is a rule for assessing the similarity and traits that will enable drug to become presumptively active medicines in humans. According to Lipinski’s rule, an orally active drug should not possess more than one violation.

Table 1 demonstrated the theoretical calculations of ADME parameters (molecular weight (MW), topological polar surface area (TPSA), partition coefficient (log P), number of hydrogen acceptors (HBA), number of hydrogen donors (HBD) and molecular volume (MV)) of the obtained compounds along with the violations of Lipinski’s rule. Acquired data stated that compounds **2a**–**2h** suited Lipinski’s rule by no violation and synthesized compounds seem to have a good pharmacokinetic profile which increases their therapeutic potentials.

Crossing the blood brain barrier (BBB) is the critical feature for the drugs specifically targeting the CNS, therefore guessing the BBB passage of novel compounds is of a great importance [16].

In respect to this, CBLigand-BBB prediction server was used to calculate BBB permeability of the compounds **2a**–**2h** [17]. This predictor practices two different algorithms as AdaBoost and Support Vector Machine (SVM), combining with four different fingerprints, employed to predict if a compound can pass (+) or cannot pass (−) the BBB. In each case, predictor scores higher than 0, if the compound can pass the BBB. As demonstrated in the Table 1, BBB values for the obtained compounds calculated as positive (+). This data especially important for the synthesized compounds as possible antidepressant agent candidates.

Following the exhibitions of good pharmacokinetic profile and sufficient BBB permeability capacities, possible antidepressant-like activities of the novel thiadiazole derivative compounds were assessed by using the tail suspension test (TST) and modified forced swimming test (MFST), two widely-used in vivo screening methods.

TST is a commonly preferred experimental model for evaluating antidepressant-like activity in rodents. This method is based on the rational that mice exposed to a temporary, unavoidable stress of being suspended by their tail, will become an immobile position. Numerous antidepressant drugs are known to reduce the immobility and increase the escape-related behaviors of animals [18].

**Figure 2:**
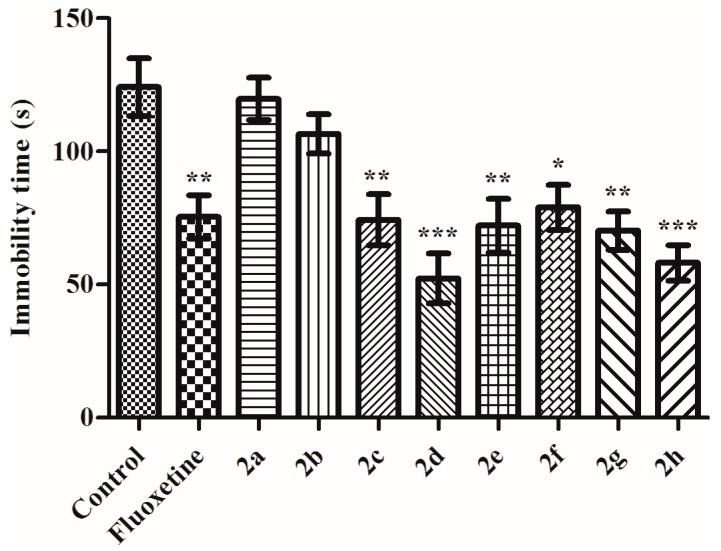
The effects of the test compounds (**2a**–**2h**) and fluoxetine on the immobility time of the mice measured in TST. Significant differences against control group, * *p* < 0.05, ** *p* < 0.01, *** *p* < 0.001. Values are presented as mean ± SEM, (*n* = 7).

In the present study, effects of the test compounds on immobility time of mice (*F* (9,69) = 8.26 *p* < 0.001) measured in the TST was demonstrated in Figure 2. Results of the post-hoc analysis indicated that administration of the compounds **2c** (*p* < 0.01), **2d** (*p* < 0.001), **2e** (*p* < 0.01), **2f** (*p* <0.05), **2g** (*p* < 0.01) and **2h** (*p* < 0.001), similar to reference drug fluoxetine (*p* < 0.01) decreased the immobility time of mice regarding to the control values. Obtained data suggested that among the tested thiadiazole derivatives **2c**, **2d**, **2e**, **2f**, **2g** and **2h** induce significant antidepressant-like effects in TST (Figure 2). Reference drug fluoxetine also show its antidepressant effect, as expected.

The forced swim test (FST) is another extensively used animal model for antidepressant-like activity screening studies. In this model, rodent is forced to swim in a water-filled cylinder and duration of the immobile posture is recorded. MFST is a modified version of classical Porsolt’s FST with increased sensitivity and specificity. In MFST, different from its traditional counterpart, active behaviors of animals, namely swimming and climbing, are also scored as well as the passive immobility behavior of animals. Reduction in the immobility as well as enhancement in the number of active behaviors are interpreted as antidepressant-like effect of the tested drug [19].

**Figure 3:**
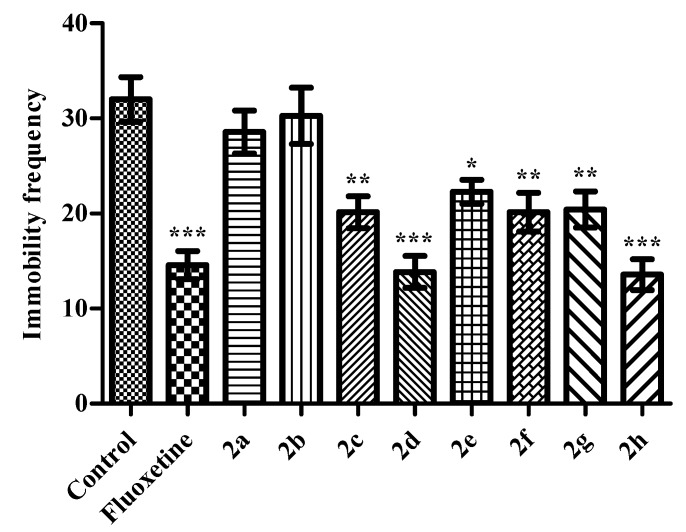
The effects of the test compounds (**2a**–**2h**) and fluoxetine on the immobility frequencies of the mice in MFST. Significant differences against control group, * *p* < 0.05, ** *p* < 0.01, *** *p* < 0.001. Values are presented as mean ± SEM, (*n* = 7).

In the MFST, effects of the test compounds on immobility frequencies of mice (*F* (9,69) = 11.65 *p* < 0.001) was shown in Figure 3. Results of the post-hoc analysis indicated that administration of the compounds **2c** (*p* < 0.01), **2d** (*p* < 0.001), **2e** (*p* < 0.05), **2f** (*p* < 0.01), **2g** (*p* < 0.01), **2h** (*p* < 0.001) and reference drug fluoxetine (*p* < 0.001) decreased the total number of immobility behaviors compared to the control values. These results supported the data obtained from the TST and evidently pointed out the antidepressant-like activities of these test compounds.

**Figure 4:**
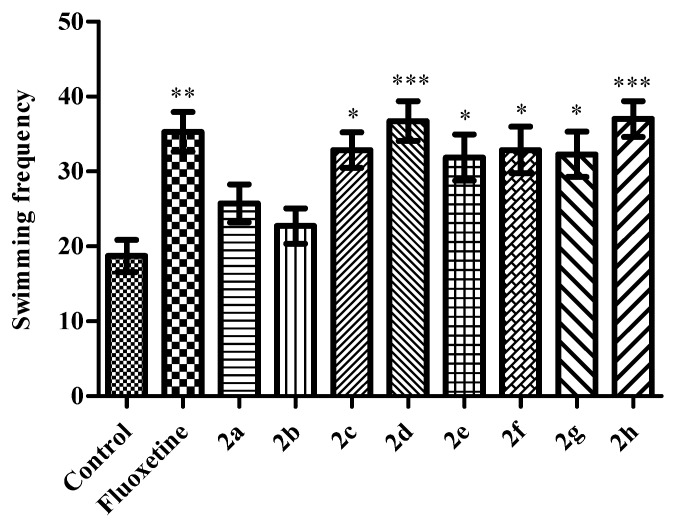
The effects of the test compounds (**2a**–**2h**) and fluoxetine on the swimming frequencies of the mice in MFST. Significant differences against control group, * *p* < 0.05, ** *p* < 0.01, *** *p* < 0.001. Values are presented as mean ± SEM, (*n* = 7).

**Figure 5:**
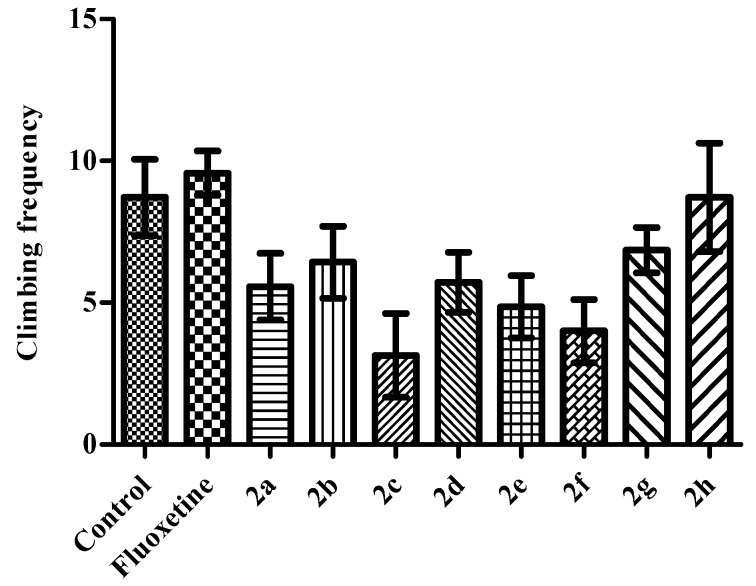
The effects of the test compounds (**2a**–**2h**) and fluoxetine on the climbing frequencies of the mice in MFST. Values are presented as mean ± SEM, (*n* = 7).

In MFST, administration of compounds **2c** (*p* < 0.05), **2d** (*p* < 0.001), **2e** (*p* < 0.05), **2f** (*p* < 0.05), **2g** (*p* < 0.05) and **2h** *p* < 0.001) induced significant enhancement in the swimming performances of mice (*F* (9,69) = 5.40 *p* < 0.001) (Figure 4), while total number of climbing behaviors were unchanged (Figure 5). Obtained findings provide us additional information related to the activity mechanism of the tested compounds. Based on the fact that serotonergic agents decrease the frequency of immobilization with simultaneous enhancement in the swimming behavior, it can be suggested that antidepressant-like activities of the test compounds observed in this study are predominantly associated to serotonergic mechanisms than catecholaminergic ones [19,20]. However, certain mechanism underlying the antidepressant-like action of these test compounds should be investigated with additional comprehensive studies.

**Figure 6:**
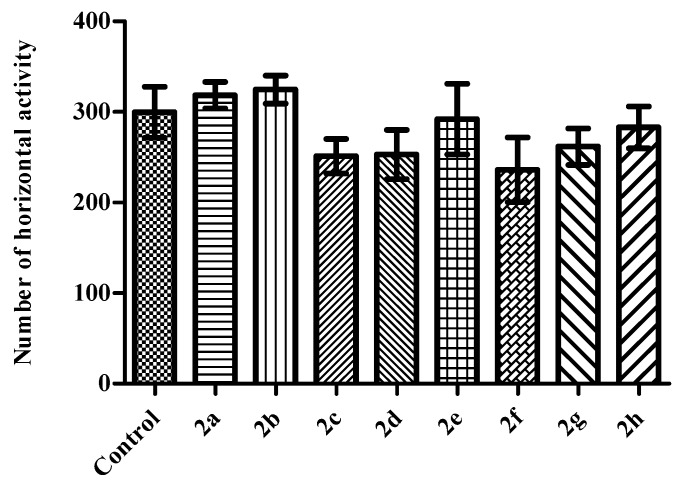
The effects of the test compounds (**2a**–**2h**) on the horizontal locomotor activities of the mice in the activity cage test. Values are presented as mean ± SEM, (*n* = 7).

**Figure 7:**
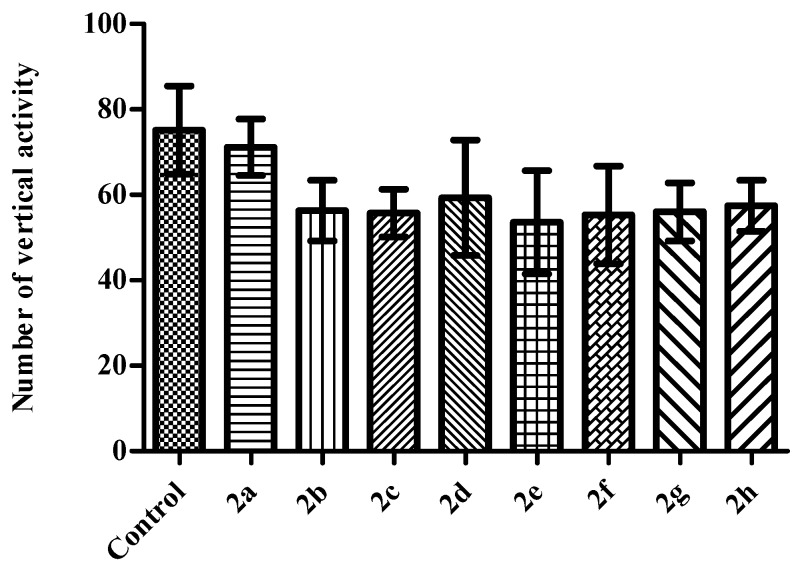
The effects of the test compounds (**2a**–**2h**) on the vertical locomotor activities of the mice in the activity cage test. Values are presented as mean ± SEM, (*n* = 7).

In the activity cage tests, compounds did not induce any alterations in the horizontal (*F* (8,62) = 1.44 *p* > 0.05) (Figure 6) or in the vertical (*F* (8,62) = 0.68 *p* > 0.05) (Figure 7) locomotor activities of the mice indicating that motor performance of the animals did not affected by the test compounds. These results exhibited that observed antidepressant-like activities are specific and not interfered with any motor impairments.

Results of this study indicated that, among the tested thiadiazole derivatives, compound **2a** and **2b** were ineffective. In the context of structure activity relationship, the possible reason of this may explained by comparing the structural differences of the synthesized compounds. Loss of antidepressant-like activity for the compounds **2a** and **2b** may be related to simultaneous substitutions of piperazine (4th position) and thiadiazole (2nd position) rings with small methyl and ethylthio groups. Actually, compounds **2d** and **2h**, substituted by bulky and hydrophobic phenyl groups on their piperazine rings, seem to be more active than the other active compounds **2c**, **2e**, **2f** and **2g**, unless the difference could not reach the statistical significance. As a matter of fact, highest log P scores of these two compounds in the serial confirmed this idea, since log P value is a predictive indicator of lipophilicity and membrane penetration.

Finally, it should be underlined that, in the studied serial, compounds possessing antidepressant-like activities exhibited negligible toxicity. None of these compounds induce death or undesirable adverse effects such as diarrhea, convulsions, ataxia, or paralysis at the administered dose. These observation is also important, since safety is one of the main parameters increasing the pharmaceutical value of novel compounds.

## 3. Materials and Methods

### 3.1. Chemistry

All reagents were purchased from commercial suppliers and were used without further purification. Prominence HPLC system (Shimadzu, Kyoto, Japan), equipped with a Shimadzu DGU-14 A degasser, LC-20 A dual piston pump, CTO-10 ASVP column oven and SPD-MI20A PDA detector Sıl-20ACXR autosampler and a stainless-steel GL Science Inertsil ODS-3 (4.6 250 mm) column. Solvents (acetonitrile and water, 7:3) for the separation obtained compounds were isocritically mixed at a flow rate of 0.25 mL/min and injection were performed by volume of 10 µL. IR spectra was recorded on an IR Affinity-*1S* Infrared spectrophotometer (Shimadzu, Tokyo, Japan). Melting points (M.p.) were determined using the Mettler Toledo-MP90 Melting Point System and were uncorrected. ^1^H NMR and ^13^C NMR spectra was recorded on a Bruker Fourier 300 (Bruker Bioscience, Billerica, MA, USA) respectively, in DMSO-*d*_6_. (Bruker, Billerica, MA, USA), respectively. MS studies were performed on an LCMS-8040 tandem mass system (Shimadzu, Tokyo, Japan).

#### 3.1.1. Synthesis of 2-Chloro-*N*-(5-methyl/ethyl-1,3,4-thiadiazol-2-yl)acetamide (**1a**, **1b**)

Chloroacetyl chloride (0.019 mol, 1.489 mL) was added dropwise with stirring to a solution of 5-methyl/ethylthio-1,3,4-thiadiazol-2-amine (0.017 mol) and trimethylamine (0.020 mol, 2.886 mL) in THF (50 mL). The mixture is stirred at 0 °C. After the completion of dropping, the mixture was allowed to stir for 1 h. Solvent was removed under reduced pressure after the reaction was complete. The residue was washed with water in an attempt to remove trimethylamine hydrochloride, then dried and recrystallized from EtOH.

#### 3.1.2. Synthesis of 2-(4-*Substituepiperazin*-1-yl)-*N*-(5-methyl/ethyl-1,3,4-thiadiazol-2-yl)acetamide (**2a**–**2h**)

A mixture of 2-chloro-*N*-(5-methyl/ethyl-1,3,4-thiadiazol-2-yl)acetamide (1a or 1b) (2.6 mmol), appropriate piperazine derivatives (2.6 mmol) and potassium carbonate (2.6 mmol, 0.363 g) as a catalytic agent were stirred in acetone (100 mL) for 5 h at 25 °C. The mixture was filtered to remove potassium carbonate and acetone was evaporated. The residue was recrystallized from EtOH.

*N-(5-Methyl-1,3,4-thiadiazol-2-yl)-2-(4-methylpiperazin-1-yl)acetamide* (**2a**). Yield: 78%, M.P. = 155–157 °C, HPLC: >%99.9. FTIR (ATR, cm^−1^): 3282 (N–H), 2943 (C–H), 1705 (C=O). ^1^H-NMR (300 MHz, DMSO-*d*_6_): δ = 2.15 (3H, s, –CH_3_), 2.32 (4H, br.s., piperazine), 2.50–2.52 (4H, m, piperazine), 2.60 (3H, s, –CH_3_), 3.29 (2H, s, –CH_2_–). ^13^C-NMR (75 MHz, DMSO-*d*_6_): δ = 15.23, 46.21, 52.90, 55.03, 60.53, 158.60, 159.71, 169.02. ESI-MS (*m*/*z*): [M + H]^+^: 256 (100%) [21].

*2-(4-Ethylpiperazin-1-yl)-N-(5-methyl-1,3,4-thiadiazol-2-yl)acetamide* (**2b**). Yield: 75%, M.P. = 107–110 °C, HPLC: >%99.9. FTIR (ATR, cm^−1^): 3354 (N–H), 2800 (C–H), 1707 (C=O). ^1^H-NMR (300 MHz, DMSO-*d*_6_): δ = 0.98 (3H, t, *J* = 8.5 Hz, –CH_3_), 2.30 (2H, q, *J* = 7.2 Hz, –CH_2_–), 2.38 (4H, br.s., piperazine), 2.50–2.53 (4H, m, piperazine), 2.61 (3H, s, –CH_3_), 3.30 (2H, s, –CH_2_–). ^13^C-NMR (75 MHz, DMSO-*d*_6_): δ = 12.46, 15.22, 52.04, 52.69, 53.05, 60.57, 158.56, 159.71, 168.99. ESI-MS (*m*/*z*): [M + H]^+^: 270 (100%).

*2-(4-Isopropylpiperazin-1-yl)-N-(5-methyl-1,3,4-thiadiazol-2-yl)acetamide* (**2c**). Yield: 80%, M.P. = 103–105 °C, HPLC: >%99.9. FTIR (ATR, cm^−1^): 3329 (N–H), 2962 (C–H), 1705 (C=O). ^1^H-NMR (300 MHz, DMSO-*d*_6_): δ = 0.96 (6H, d, *J* = 6.5 Hz, –CH_3_), 2.45–2.46 (5H, m, piperazine, -CH-), 2.50–2.54 (4H, m, piperazine), 2.61 (3H, s, –CH_3_), 3.28 (2H, s, –CH_2_–). ^13^C-NMR (75 MHz, DMSO-*d*_6_): δ = 15.23, 18.68, 48.30, 53.48, 54.05, 60.63, 158,54, 159.73, 169.02. ESI-MS (*m*/*z*): [M + H]^+^: 284 (100%).

*N-(5-Methyl-1,3,4-thiadiazol-2-yl)-2-(4-phenylpiperazin-1-yl)acetamide* (**2d**). Yield: 83%, M.P. = 155–158 °C, HPLC: %93.4. FTIR (ATR, cm^−1^): 3282 (N–H), 2943 (C–H), 1705 (C=O). 660, 750. ^1^H-NMR (300 MHz, DMSO-*d*_6_): δ = 2.61 (3H, s, –CH_3_), 2.65–2.69 (4H, m, piperazine), 3.14–3.18 (4H, m, piperazine), 2.49–2.52 (4H, m, piperazine), 3.39 (2H, s, –CH_2_–). 6.77 (1H, t, *J* = 7.2 Hz, phenyl), 6.92 (2H, d, *J* = 8.7 Hz, phenyl), 7.21 (2H, t, *J* = 8.0 Hz, phenyl). ^13^C-NMR (75 MHz, DMSO-*d*_6_): δ = 15.22, 48.63, 52.91, 60.46, 115.91, 119.29, 129.37, 151.46, 158.56, 159.77, 168.93. ESI-MS (*m*/*z*): [M + H]^+^: 318 (100%).

*N-(5-(Ethylthio)-1,3,4-thiadiazol-2-yl)-2-(4-methylpiperazin-1-yl)acetamide* (**2e**). Yield: 79%, oil, HPLC: %94.4. FTIR (ATR, cm^−1^): 3329 (N–H), 2937 (C–H), 1699 (C=O). ^1^H-NMR (300 MHz, DMSO-*d*_6_): δ = 1.27 (3H, t, *J* = 7.2 Hz, –CH_3_), 2.13 (3H, s, –CH_3_), 2.29 (4H, br.s., piperazine), 2.45 (4H, br.s., piperazine), 3.01 (2H, q, *J* = 7.3 Hz, –CH_2_–), 2.99 (2H, s, –CH_2_–). ^13^C-NMR (75 MHz, DMSO-*d*_6_): δ = 15.55, 28.39, 46.37, 53.29, 55.33, 65.25, 167.62, 170.45, 174.81. ESI-MS (*m*/*z*): [M + H]^+^: 302 (100%).

*2-(4-Ethylpiperazin-1-yl)-N-(5-(ethylthio)-1,3,4-thiadiazol-2-yl)acetamide* (**2f**). Yield: 74%, oil, HPLC: %99.3. FTIR (ATR, cm^−1^): 3365 (N–H), 2970 (C–H), 1699 (C=O). ^1^H-NMR (300 MHz, DMSO-*d*_6_): δ = 0.97 (3H, t, *J* = 7.2 Hz, –CH_3_), 1.30 (3H, t, *J* = 7.3 Hz, –CH_3_), 2.28 (2H, q, *J* = 7.2 Hz, –CH_2_–), 2.35 (4H, br.s., piperazine), 2.48 (4H, br.s., piperazine), 3.11 (2H, q, *J* = 7.3 Hz, –CH_2_–), 3.16 (2H, s, –CH_2_–). ^13^C-NMR (75 MHz, DMSO-*d*_6_): δ = 12.52, 15.37, 28.46, 52.11, 52.82, 53.21, 62.79, 155.25, 164.47, 171.98. ESI-MS (*m*/*z*): [M + H]^+^: 316 (100%).

*N-(5-(Ethylthio)-1,3,4-thiadiazol-2-yl)-2-(4-isopropylpiperazin-1-yl)acetamide* (**2g**). Yield: 78%, oil, HPLC: %97.9. FTIR (ATR, cm^−1^): 3344 (N–H), 2960 (C–H), 1699 (C=O). ^1^H-NMR (300 MHz, DMSO-*d*_6_): δ = 0.95 (6H, d, *J* = 6.5 Hz, –CH_3_), 1.33 (3H, t, *J* = 7.3 Hz, –CH_3_), 2.44–2.45 (4H, m, piperazine), 2.48–2.51 (4H, m, piperazine), 2.55–2.64 (1H, m, –CH–), 3.19 (2H, q, *J* = 7.3 Hz, –CH_2_–), 3.26 (2H, s, –CH_2_–). ^13^C-NMR (75 MHz, DMSO-*d*_6_): δ = 15.25, 18.69, 28.50, 48.28, 53.47, 54.06, 61.15, 157.92, 160.43, 170.05. ESI-MS (*m*/*z*): [M + H]^+^: 330 (100%).

*N-(5-(Ethylthio)-1,3,4-thiadiazol-2-yl)-2-(4-phenylpiperazin-1-yl)acetamide* (**2h**). Yield: 85%, M.P. = 152–154 °C, HPLC: %95.8. FTIR (ATR, cm^−1^): 3373 (N–H), 2960 (C–H), 1699 (C=O), 688, 756. ^1^H-NMR (300 MHz, DMSO-*d*_6_): δ = 1.33 (3H, t, *J* = 7.2 Hz, –CH_3_), 2.65–2.68 (4H, m, piperazine), 3.13–3.16 (4H, m, piperazine), 2.45 (4H, br.s., piperazine), 3.20 (2H, q, *J* = 7.3 Hz, –CH_2_–), 3.37 (2H, s, –CH_2_–), 6.77 (1H, t, *J* = 7.2 Hz, phenyl), 6.92 (2H, d, *J* = 7.9 Hz, phenyl), 7.20 (2H, t, *J* = 7.9 Hz, phenyl). ^13^C-NMR (75 MHz, DMSO-*d*_6_): δ = 15.25, 28.52, 48.62, 52.92, 60.86, 115.89, 119.26, 129.37, 151.48, 158.22, 160.07, 169.84. ESI-MS (*m*/*z*): [M + H]^+^: 364 (100%).

### 3.2. Prediction of ADME Parameters and BBB Permeability

Predictions of ADME properties of compounds **2a**–**2h** were calculated by means of Online Molinspiration property calculation program [15]. The BBB permeability of the compounds was assessed by using an online BBB Predictor [17].

### 3.3. Pharmacology

#### 3.3.1. Animals

Experiments were conducted with adult male BALB/c mice weighing 30–35 g. Animals were kept on a 12 h light-dark cycle (08:00–20:00) in a silent room at 25 ± 1 °C. Temperature, sound, humidity and light conditions were the same during the tests. Food and water were given ad libitum. The experimental protocol of this study was approved by the Local Ethical Committee on Animal Experimentation of Eskişehir Anadolu University, Turkey.

#### 3.3.2. Administration of Compounds

Experimental groups were formed as follows: Control (vehicle treated group), reference drug group (fluoxetine treated group) and 8 test groups (8 different groups treating by the test compounds). Vehicle, fluoxetine (Flx, 10 mg/kg) and the test compounds (30 mg/kg) were administrated intraperitoneally (i.p.) for 3 times; 24, 5 and 0.5 h before the experiments [22].

#### 3.3.3. Behavioral Tests

##### Tail Suspension Test

Antidepressant-like efficacy of test compounds were assessed by TST, as described elsewhere [23,24]. Mice were individually secured to the metal rod 30 cm above the floor by adhesive tape placed 1 cm from the tip of the tail. The animals were assumed as immobile when they hung passively without any active behavior. During the last 4 of 6 min test period, total time of immobility behavior was recorded, manually.

##### Modified Forced Swimming Test

Antidepressant-like efficacy of the test compounds were also evaluated by MFST [20,25]. MFST was carried out in a glass cylinder (12 cm in diameter × 30 cm height) filled with 20 cm of water at 25 ± 1 °C. Animals were individually submitted to 15-min pre-test 24 h prior to 5-min swim test.

In the test period, total number of immobility (only movements required to keep the head above the water), swimming (horizontal movement on the surface of the water) and climbing (upward-directed movements with forelegs above the water level) behaviors of mice over a 5-s interval were recorded, manually.

The water was refill after each trial. Following the training and the test sessions, mice were dried in a heated enclosure before returning to home cages.

##### Activity Cage Test

Probable effects of the test compounds on spontaneous locomotor activities of animals were evaluated in an activity cage apparatus (No. 7420; Ugo Basile, Varese, Italy), containing two pairs of 16 infrared photocells. Mice were located individually in a device for 4 min and interruptions of light beams to the photocells throughout horizontal and vertical behavior of the mouse were recorded by the apparatus. After each trial, the floor of the cage was cleaned with alcohol [26,27].

##### Statistical Analyses

Statistical evaluation of data was performed using the GraphPad Prism 6.01 (GraphPad Software, San Diego, CA, USA). Data, acquired from 7 animals for each group, were analyzed using one-way analysis of variance (ANOVA) followed by Tukey’s test.

Experimental results are given as mean (s) ± standard error of the mean (S.E.M). *p* values less than 0.05 were considered to reflect a significant difference.

## 4. Conclusions

In this paper, we focused on the synthesis and pharmacological assessment of some novel thiadiazole derivatives as potential antidepressant agents.

Findings of the study exhibited anti-depressant-like efficacy of the tested thiadiazole derivative compounds and suggested serotonergic mechanisms related mode of actions. These results are in accordance with some earlier papers reporting the anti-depressant activities of chemical compounds obtained by the derivation of thiadiazole ring [4,10,11,12,13]. On the other hand, it is clear that further mechanistic researches and safety studies are needed for these molecules to offer them as new antidepressant drug candidates.

## Figures and Tables

**Table:** 

Compounds	R_1_	R_2_
**2a**	methyl	methyl
**2b**	methyl	ethyl
**2c**	methyl	isopropyl
**2d**	methyl	phenyl
**2e**	ethylthio	methyl
**2f**	ethylthio	ethyl
**2g**	ethylthio	isopropyl
**2h**	ethylthio	phenyl

**Table 1:** Some physicochemical parameters used in prediction of absorption, distribution, metabolism, excretion (ADME) profiles of the compounds **2a**–**2h** and fluoxetine.

Comp.	MW	TPSA	Log P	HBA	HBD	MV	Vio	BBB
**2a**	255.35	61.36	−0.16	6	1	229.69	0	+
**2b**	269.37	61.36	0.21	6	1	246.49	0	+
**2c**	283.40	61.36	0.51	6	1	263.07	0	+
**2d**	317.42	61.36	1.53	6	1	284.53	0	+
**2e**	301.44	61.36	1.22	6	1	264.62	0	+
**2f**	315.47	61.36	1.59	6	1	281.42	0	+
**2g**	329.50	61.36	1.89	6	1	298.00	0	+
**2h**	363.51	61.36	2.92	6	1	319.46	0	+
**Fluoxetine**	309.33	21.26	4.53	2	1	275.13	0	+

## Supplementary Materials

Supplementary File 1
