# Supplementary material for: Synthesis of Some Novel Thiadiazole Derivative Compounds and Screening Their Antidepressant-Like Activities
Source: Molecules. 2018 Mar 15;23(4):716. doi: 10.3390/molecules23040716 (PMC6017710; doi:10.3390/molecules23040716)
Supplement: Supplementary File 1 [file molecules-23-00716-s001.pdf]

DOPNALAB

	Item	Value
	Acquired Date&Time	13.12.2017 08:56:19
	Acquired by	System Administrator
	Filename	C:\Users\dopnalab\Desktop\derya\T serisi\T-12.ispd
	Spectrum name	T-12
	Sample name	T-1
	Sample ID	
	Option	
	Comment	
	No. of Scans	10
	Resolution	4 [cm-1]
	Apodization	Happ-Genzel

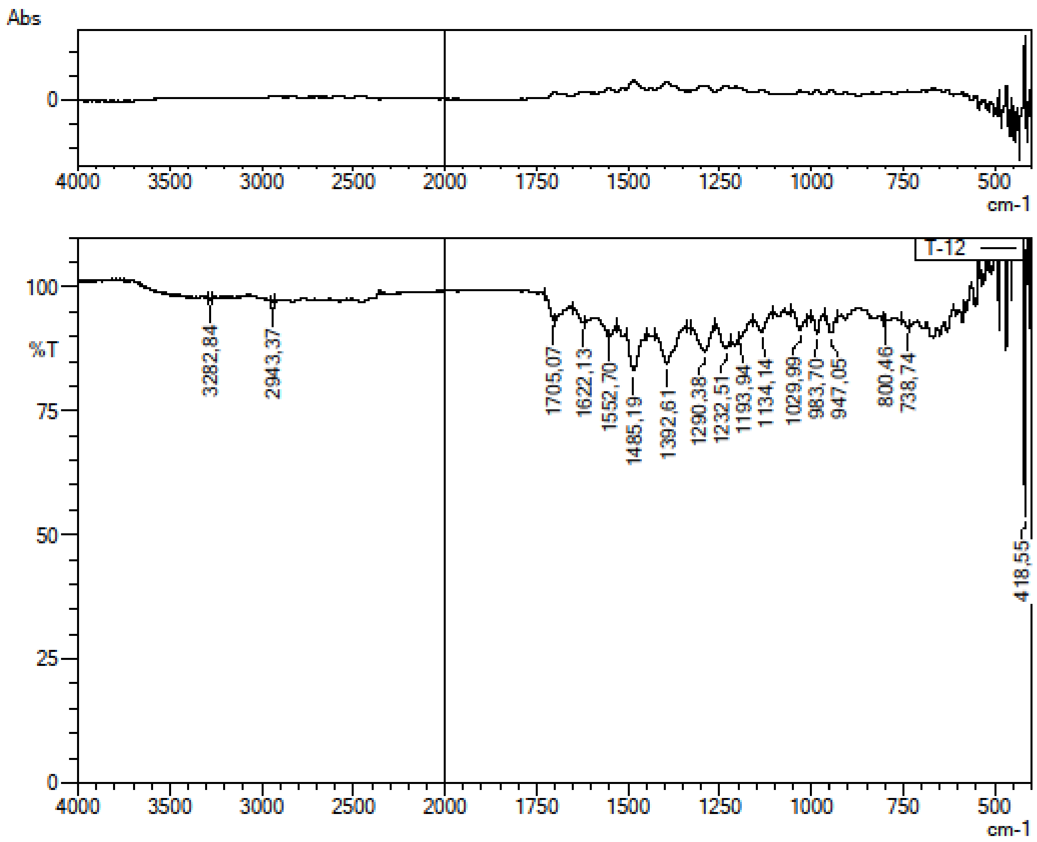

Spectra 1. IR spectra of compound **2a**

LCMSMS ANALYSES REPORT

Sample Name	: T-1		
Sample ID	:		
Data Filename	: T-1_002.lcd		
Method Filename	: genel.lcm		
Batch Filename	:GENEL_batch_tarama.lcb		
Vial #	: 1-16		
Injection Volume	: 1 uL	Sample Type	: Unknown
Date Acquired	: 19.10.2017 14:45:07	Acquired by	: System Administrator
Date Processed	: 19.10.2017 14:47:10	Processed by	: System Administrator

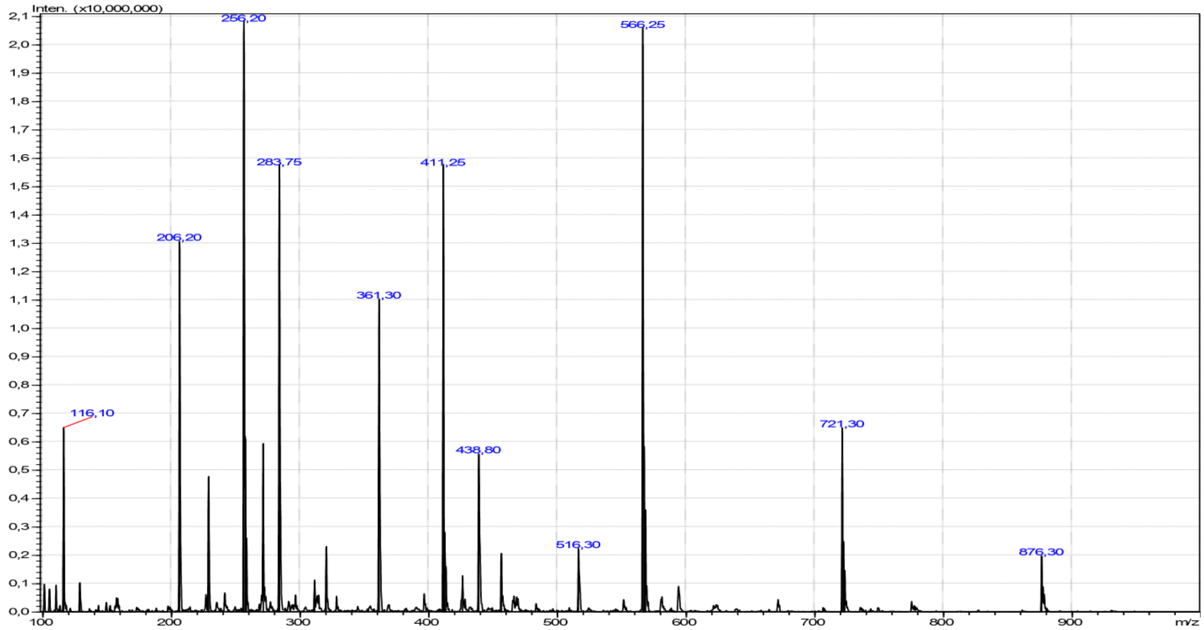

[MS Spectrum]
# of Peaks 26
Raw Spectrum [0,203->0,203],(scan:[13->13])
Background [1,015->1,827],(scan:[61->109])
Base Peak m/z 256,20 (Inten : 20.000.000)

Spectra 2. LCMSMS spectra of compound **2a**

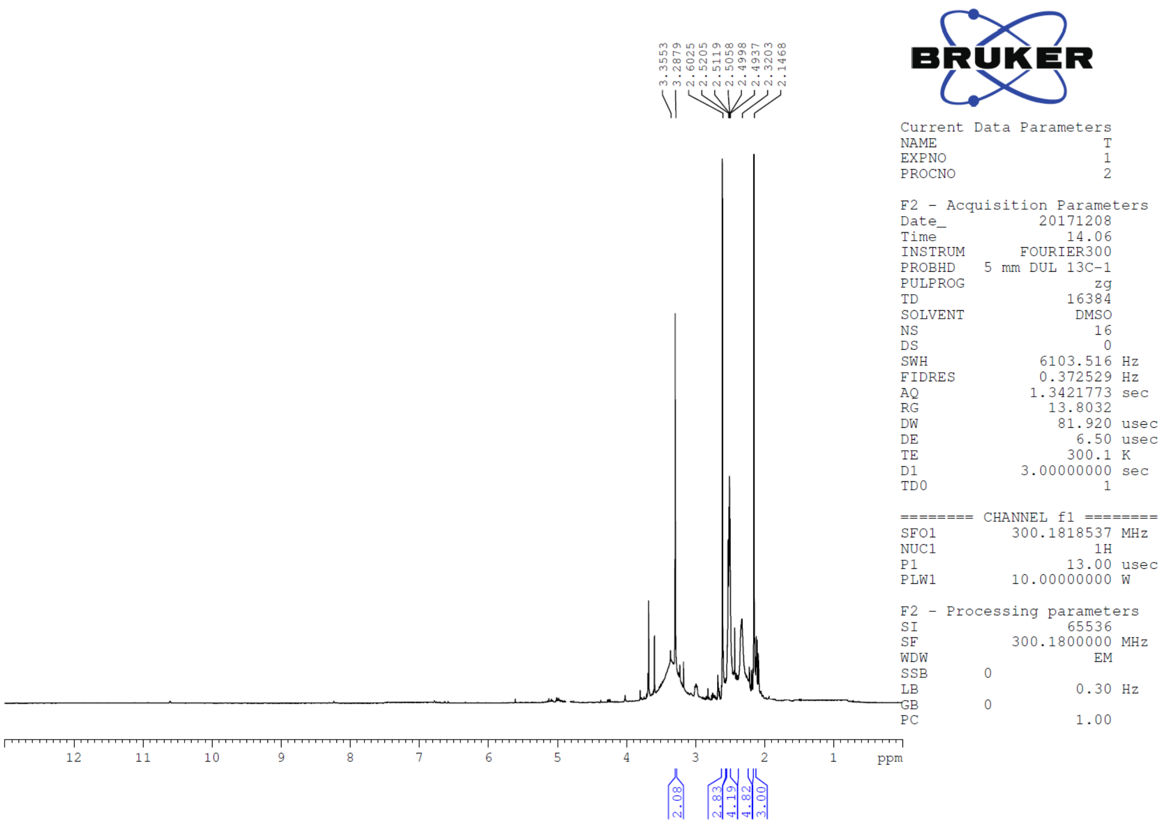

Spectra 3. ^{1}H-NMR spectra of compound **2a**

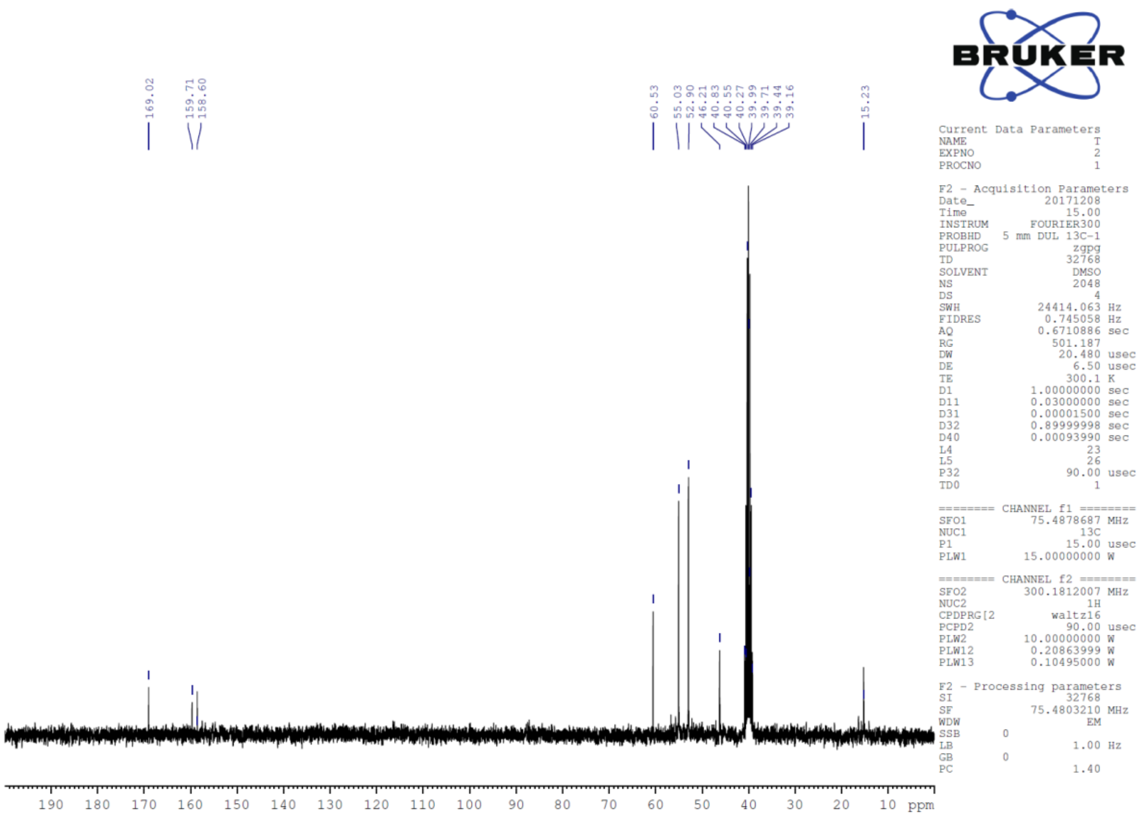

Spectra 4. ^{13}C-NMR spectra of compound **2a**

DOPNALAB

	Item	Value
	Acquired Date&Time	13.12.2017 09:01:25
	Acquired by	System Administrator
	Filename	C:\Users\dopnalab\Desktop\derya\T serisi\T-21.ispd
	Spectrum name	T-21
	Sample name	T-2
	Sample ID	
	Option	
	Comment	
	No. of Scans	10
	Resolution	4 [cm-1]
	Apodization	Happ-Genzel

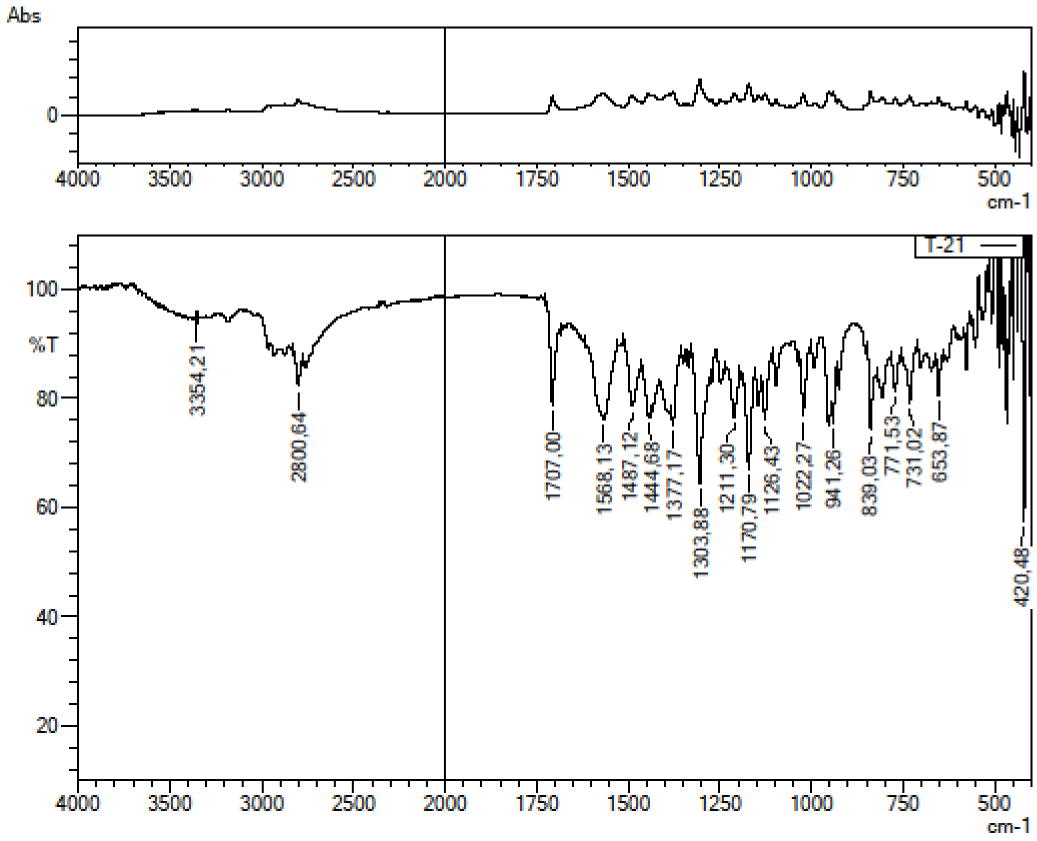

Spectra 5. IR spectra of compound **2b**

LCMSMS ANALYSES REPORT

Sample Name	: T-2		
Sample ID	:		
Data Filename	: T-2_003.lcd		
Method Filename	: genel.lcm		
Batch Filename	:GENEL_batch_tarama.lcb		
Vial #	: 1-17		
Injection Volume	: 1 uL	Sample Type	: Unknown
Date Acquired	: 19.10.2017 14:47:17	Acquired by	:System Administrator
Date Processed	: 19.10.2017 14:49:20	Processed by	:System Administrator

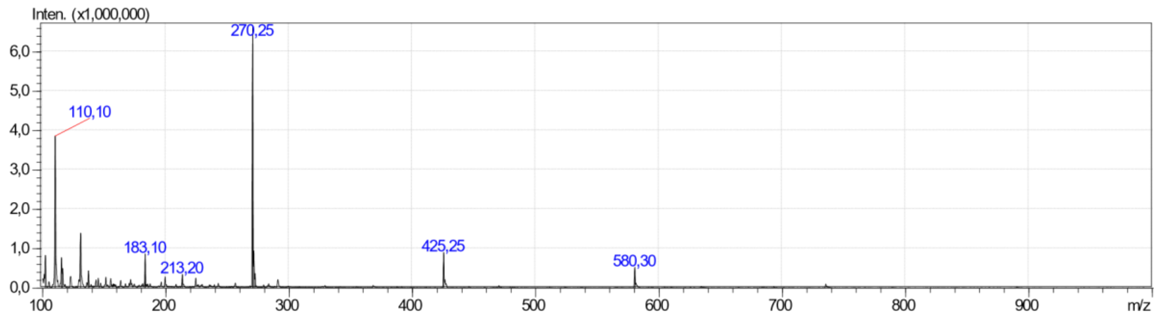

[MS Spectrum]

# of Peaks 13

Raw Spectrum [0,575->0,711],(scan:[35->43])

Background No Background Spectrum

Base Peak m/z 270,25 (Inten : 6.616.908)

Spectra 6. LCMSMS spectra of compound **2b**

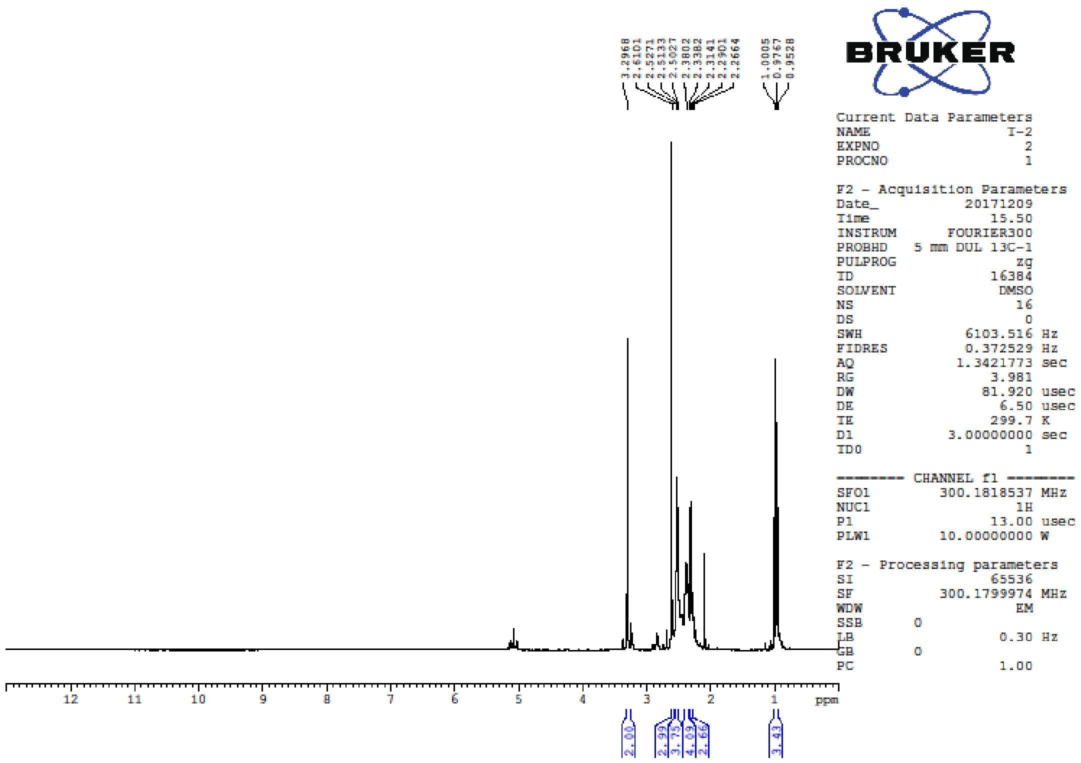

Spectra 7. ^{1}H-NMR spectra of compound **2b**

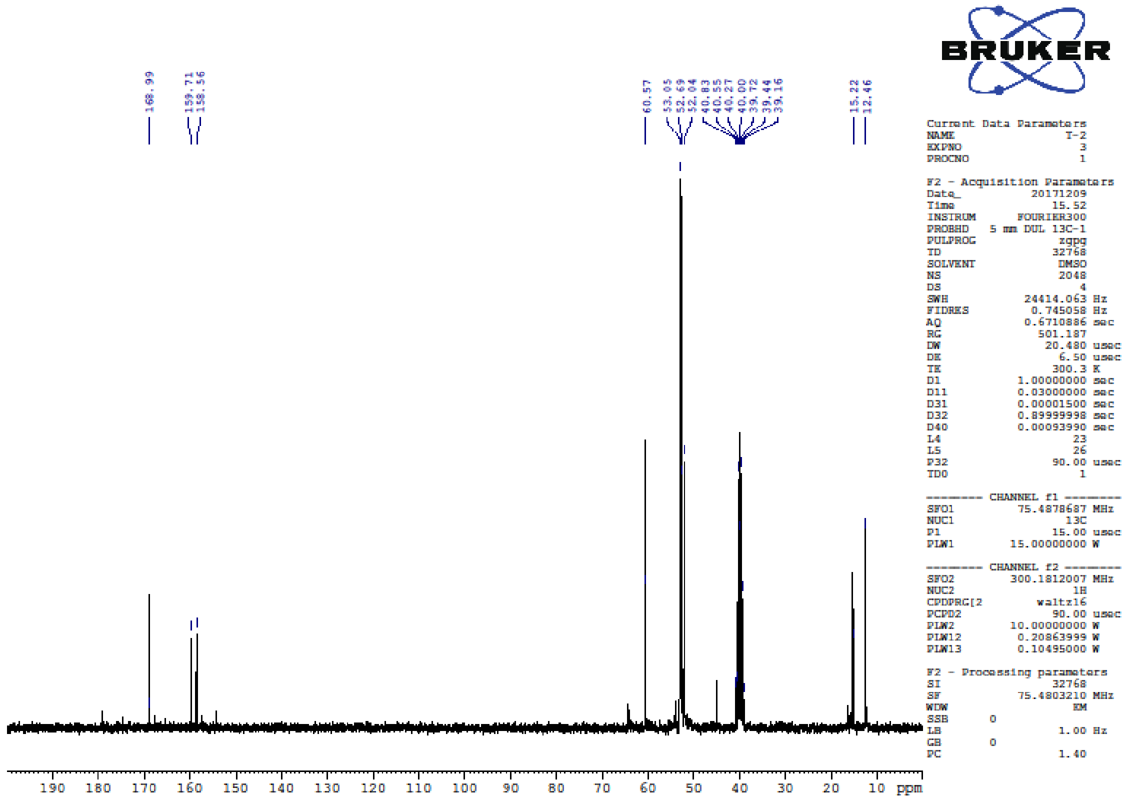

Spectra 8. ^{13}C-NMR spectra of compound **2b**

DOPNALAB

Item	Value
Acquired Date&Time	13.12.2017 09:03:16
Acquired by	System Administrator
Filename	C:\Users\dopnalab\Desktop\derya\T serisi\T-31.ispd
Spectrum name	T-31
Sample name	T-3
Sample ID	
Option	
Comment	
No. of Scans	10
Resolution	4 [cm-1]
Apodization	Happ-Genzel

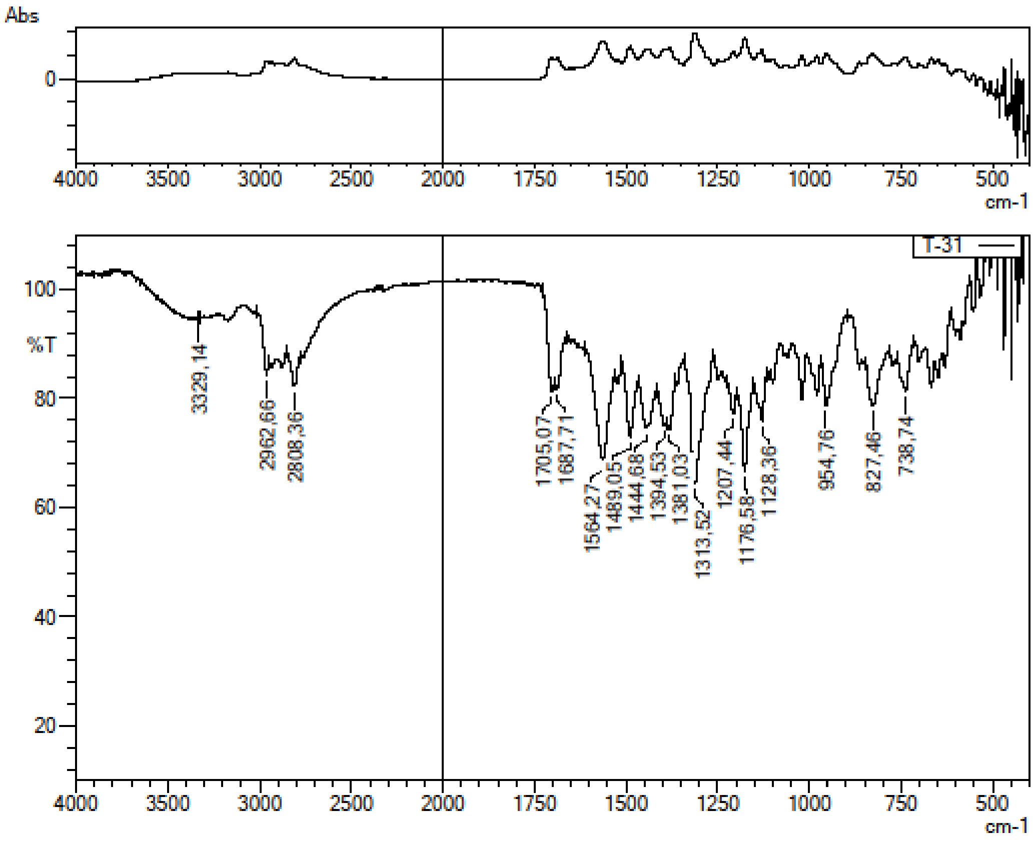

Spectra 9. IR spectra of compound **2c**

LCMSMS ANALYSES REPORT

Sample Name	: T-3		
Sample ID	:		
Data Filename	: T-3_004.lcd		
Method Filename	: genel.lcm		
Batch Filename	:GENEL_batch_tarama.lcb		
Vial #	: 1-18		
Injection Volume	: 1 uL	Sample Type	: Unknown
Date Acquired	: 19.10.2017 14:49:30	Acquired by	:System Administrator
Date Processed	: 19.10.2017 14:51:40	Processed by	:System Administrator

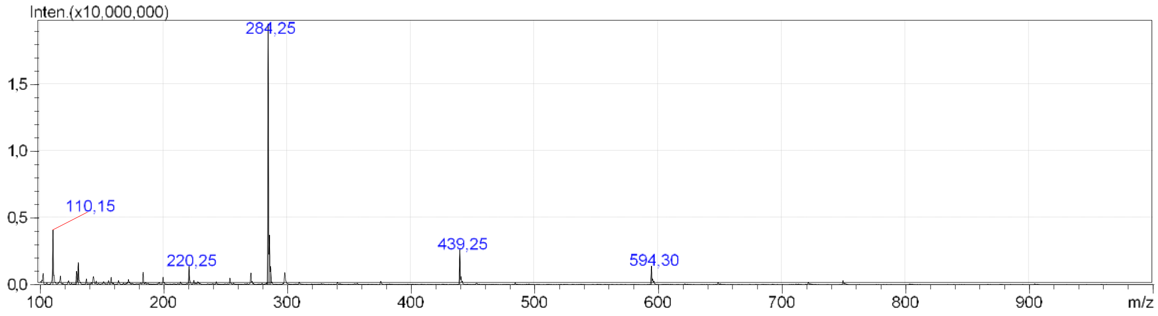

[MS Spectrum]

# of Peaks 9

Raw Spectrum [0,372->0,474],(scan:[23->29])

Background No Background Spectrum

Base Peak m/z 284,25 (Inten : 19.488.994)

Spectra 10. LCMSMS spectra of compound **2c**

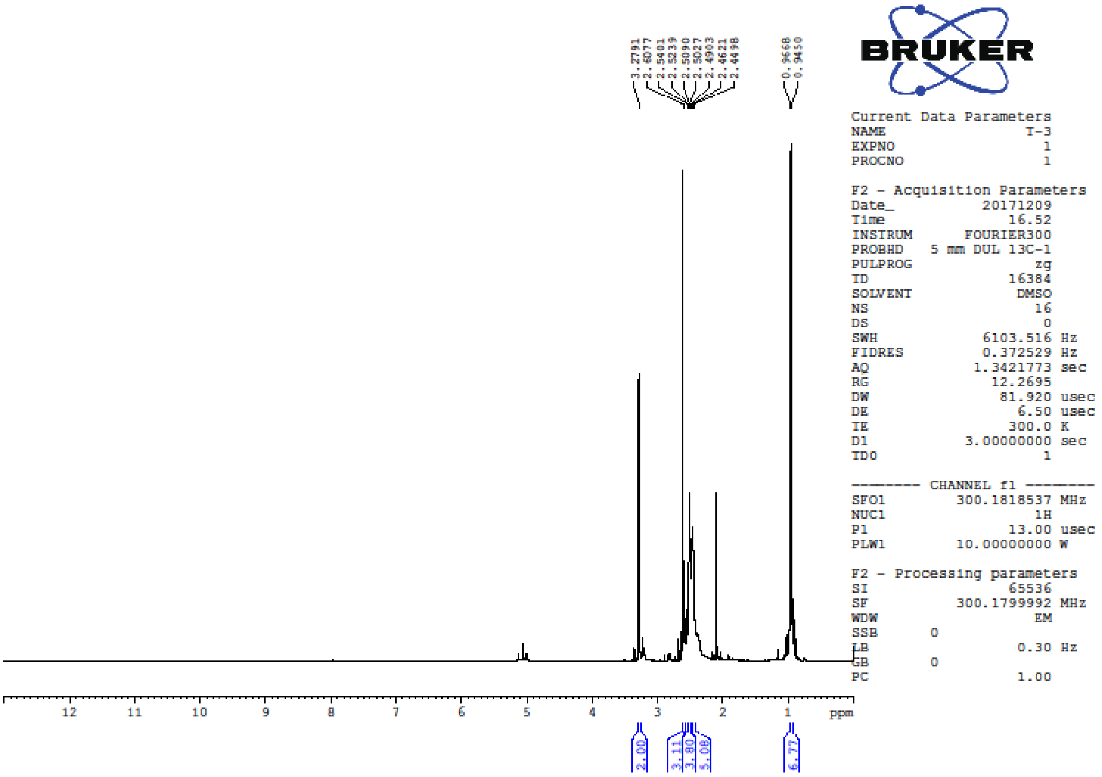

Spectra 11. ^{1}H-NMR spectra of compound **2c**

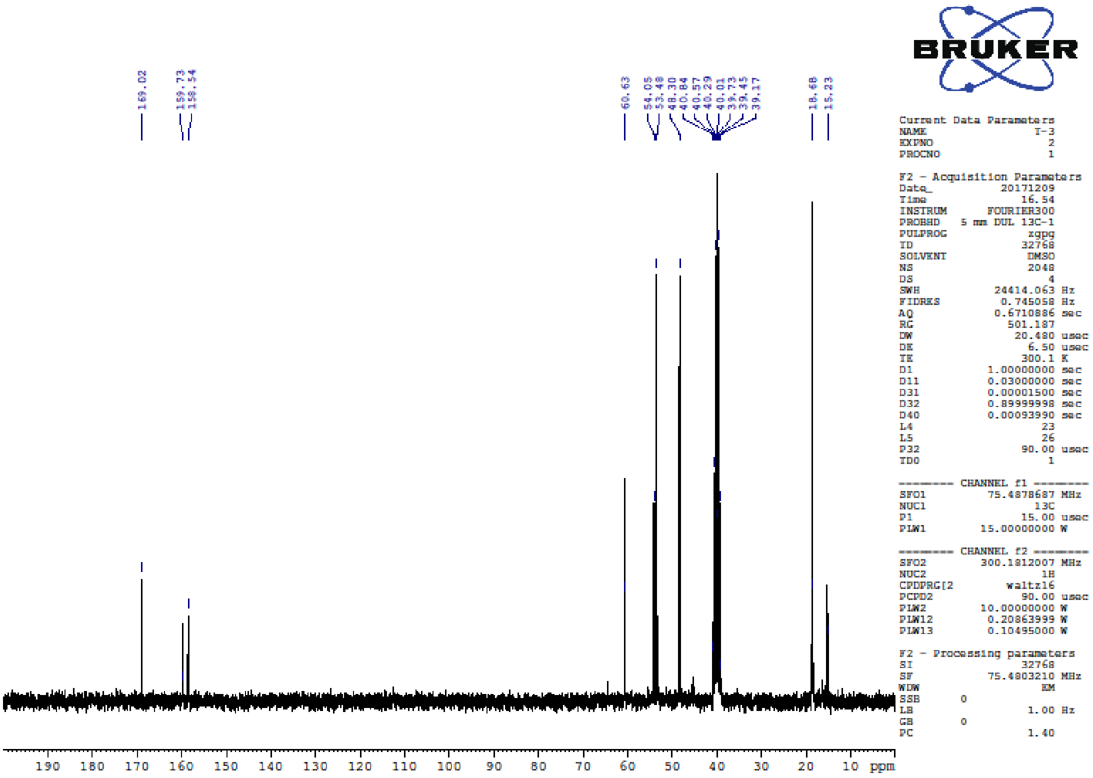

Spectra 12. ^{13}C-NMR spectra of compound **2c**

DOPNALAB

	Item	Value
	Acquired Date&Time	13.12.2017 09:04:36
	Acquired by	System Administrator
	Filename	C:\Users\dopnalab\Desktop\derya\T serisi\T-41.ispd
	Spectrum name	T-41
	Sample name	T-4
	Sample ID	
	Option	
	Comment	
	No. of Scans	10
	Resolution	4 [cm-1]
	Apodization	Happ-Genzel

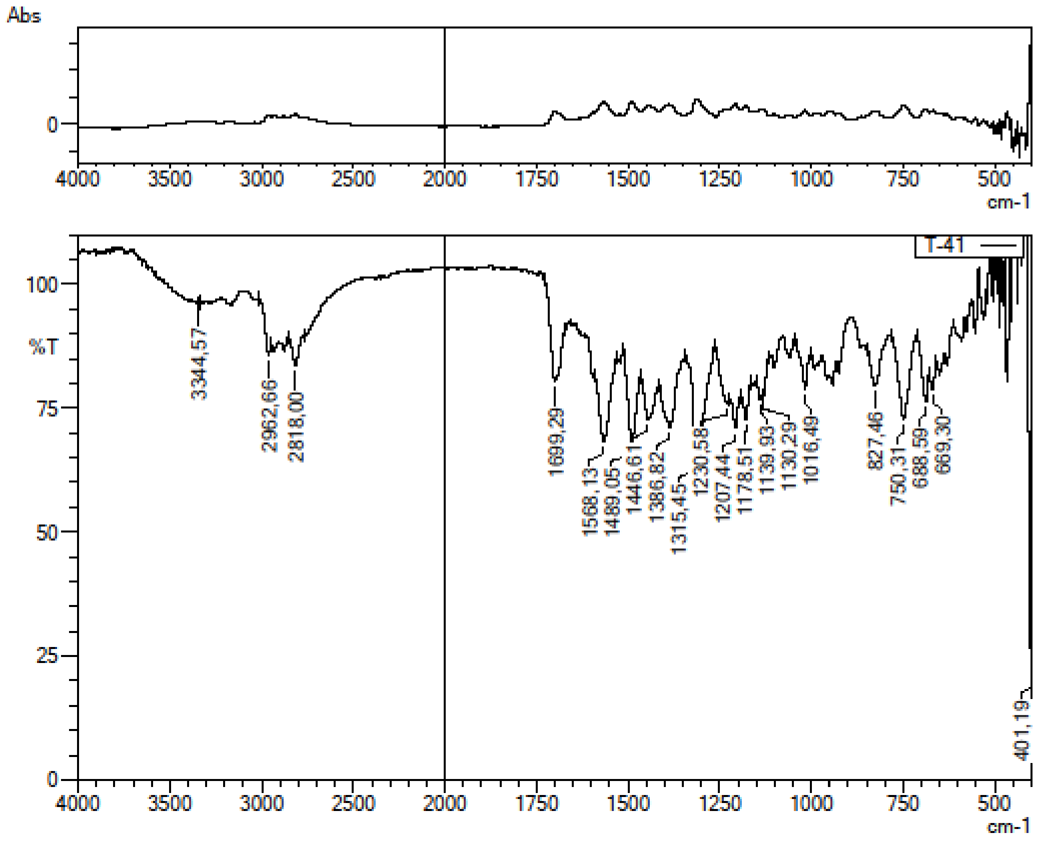

Spectra 13. IR spectra of compound **2d**

LCMSMS ANALYSES REPORT

Sample Name	: T-4		
Sample ID	:		
Data Filename	: T-4_005.lcd		
Method Filename	: genel.lcm		
Batch Filename	:GENEL_batch_tarama.lcb		
Vial #	: 1-19		
Injection Volume	: 1 uL	Sample Type	: Unknown
Date Acquired	: 19.10.2017 14:51:50	Acquired by	:System Administrator
Date Processed	: 19.10.2017 14:54:03	Processed by	:System Administrator

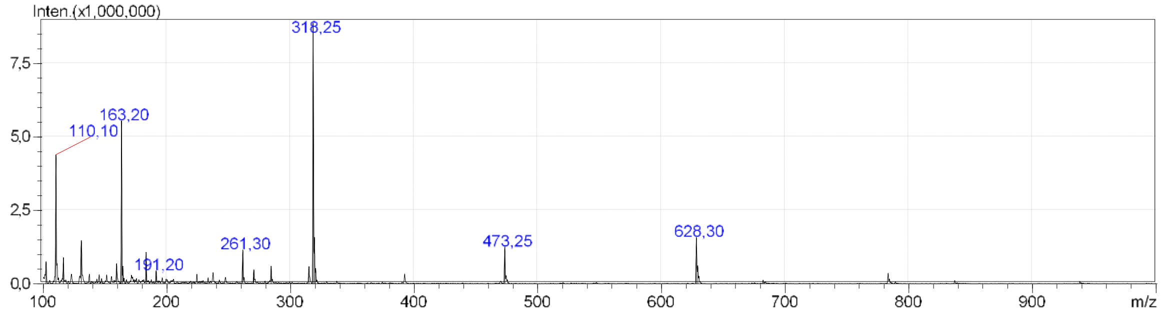

[MS Spectrum]

# of Peaks 19

Raw Spectrum [0,541->0,609],(scan:[33->37])

Background No Background Spectrum

Base Peak m/z 318,25 (Inten : 8.835.278)

Spectra 14. LCMSMSspectra of compound **2d**

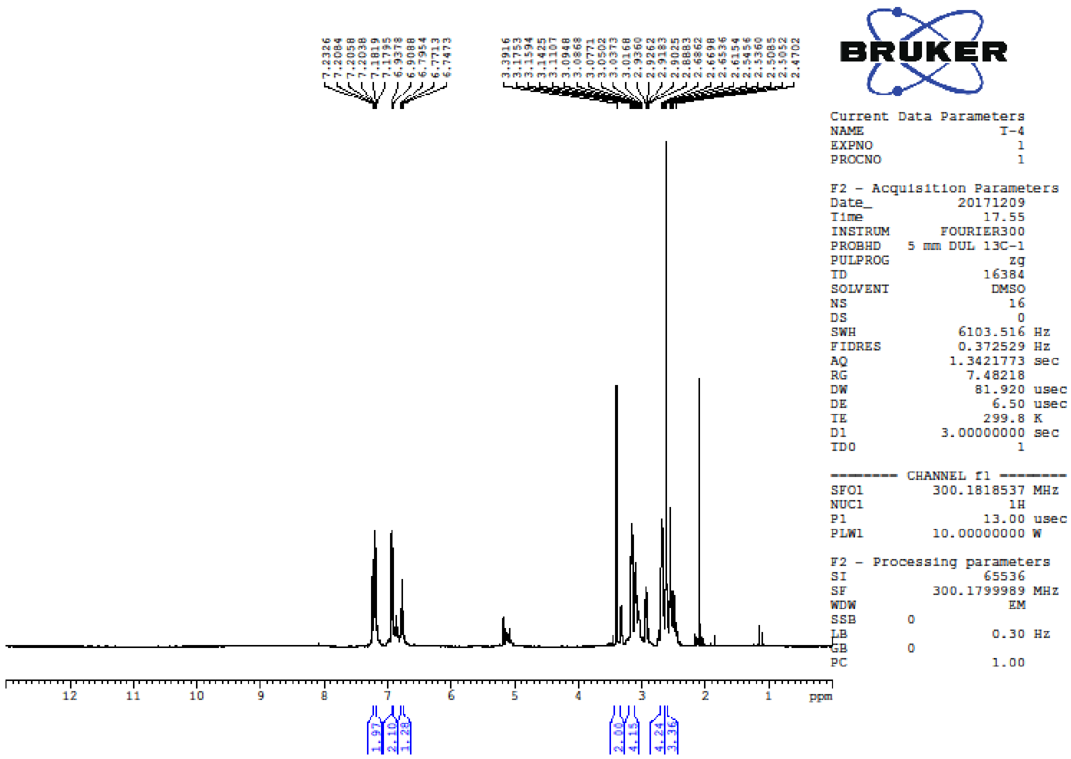

Spectra 15. ^{1}H-NMR spectra of compound **2d**

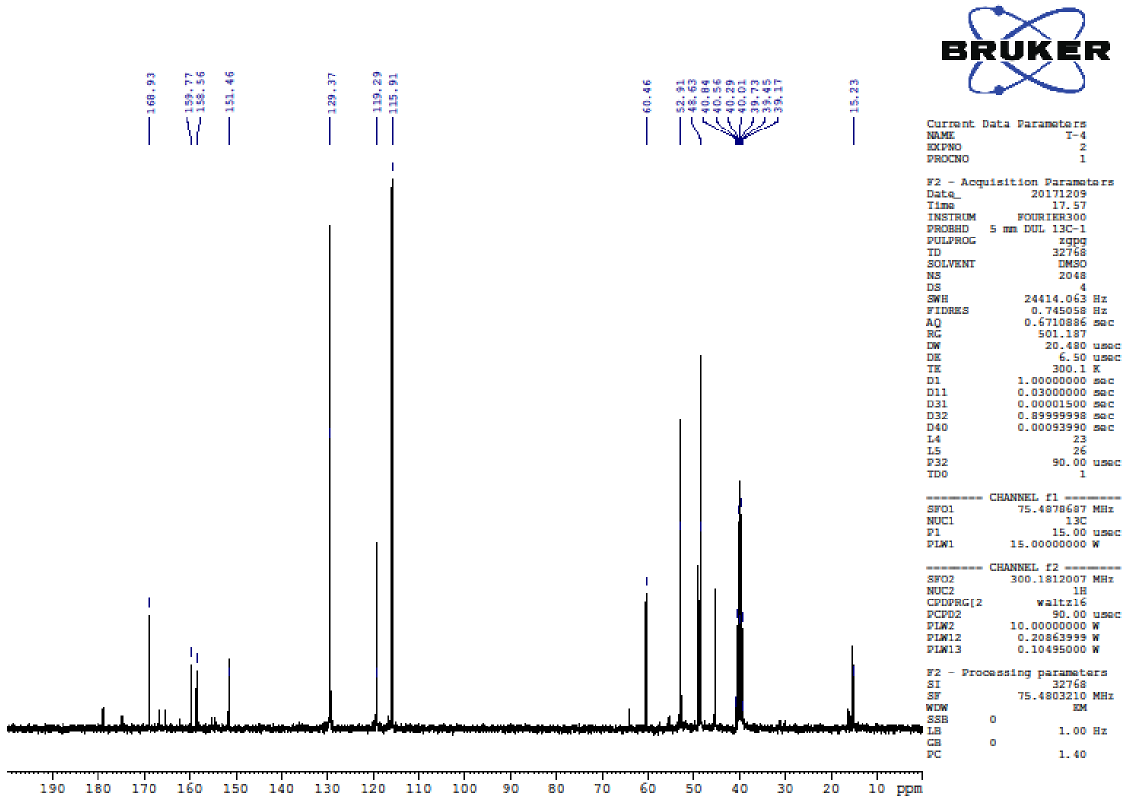

Spectra 16. ^{13}C-NMR spectra of compound **2d**

DOPNALAB

Item	Value
Acquired Date&Time	13.12.2017 09:10:50
Acquired by	System Administrator
Filename	C:\Users\dopnalab\Desktop\derya\T serisi\T-51.ispd
Spectrum name	T-51
Sample name	T-5
Sample ID	
Option	
Comment	
No. of Scans	10
Resolution	4 [cm-1]
Apodization	Happ-Genzel

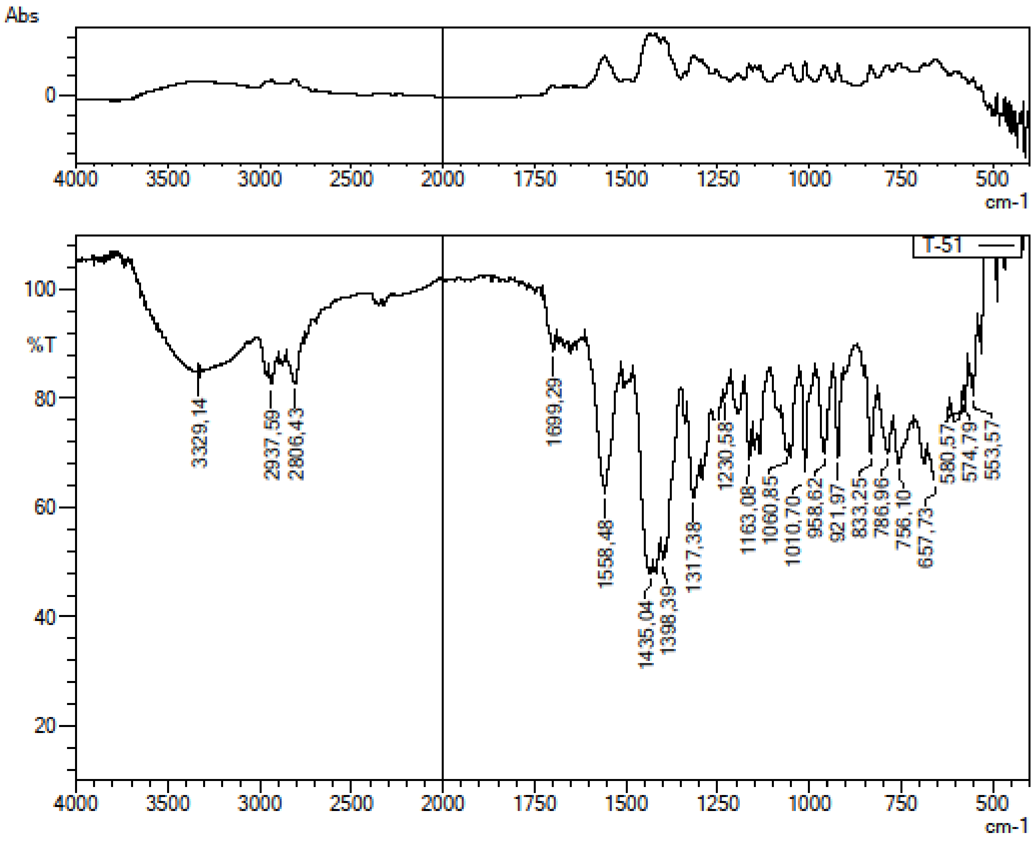

Spectra 17. IR spectra of compound **2e**

LCMSMS ANALYSES REPORT

Sample Name	: T-5		
Sample ID	:		
Data Filename	: T-5_006.lcd		
Method Filename	: genel.lcm		
Batch Filename	:GENEL_batch_tarama.lcb		
Vial #	: 1-20		
Injection Volume	: 1 uL	Sample Type	: Unknown
Date Acquired	: 19.10.2017 14:54:13	Acquired by	:System Administrator
Date Processed	: 19.10.2017 14:56:25	Processed by	:System Administrator

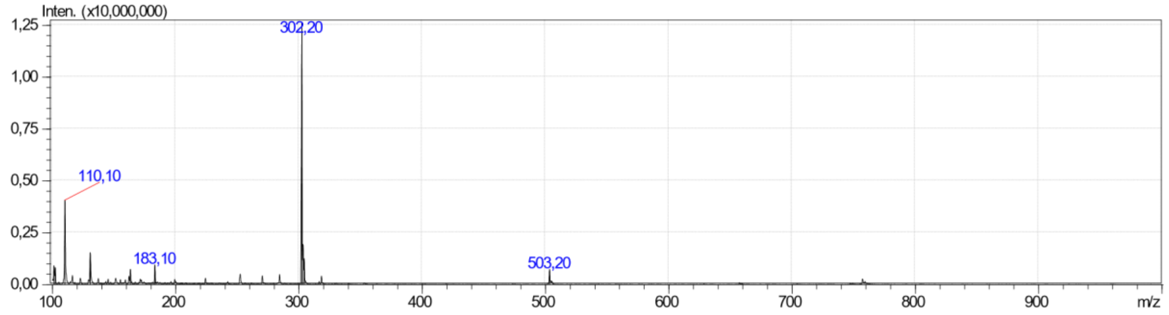

[MS Spectrum]

# of Peaks 10

Raw Spectrum [0,406->0,541],(scan:[25->33])

Background No Background Spectrum

Base Peak m/z 302,20 (Inten : 12.580.969)

Spectra 18. LCMSMS spectra of compound **2e**

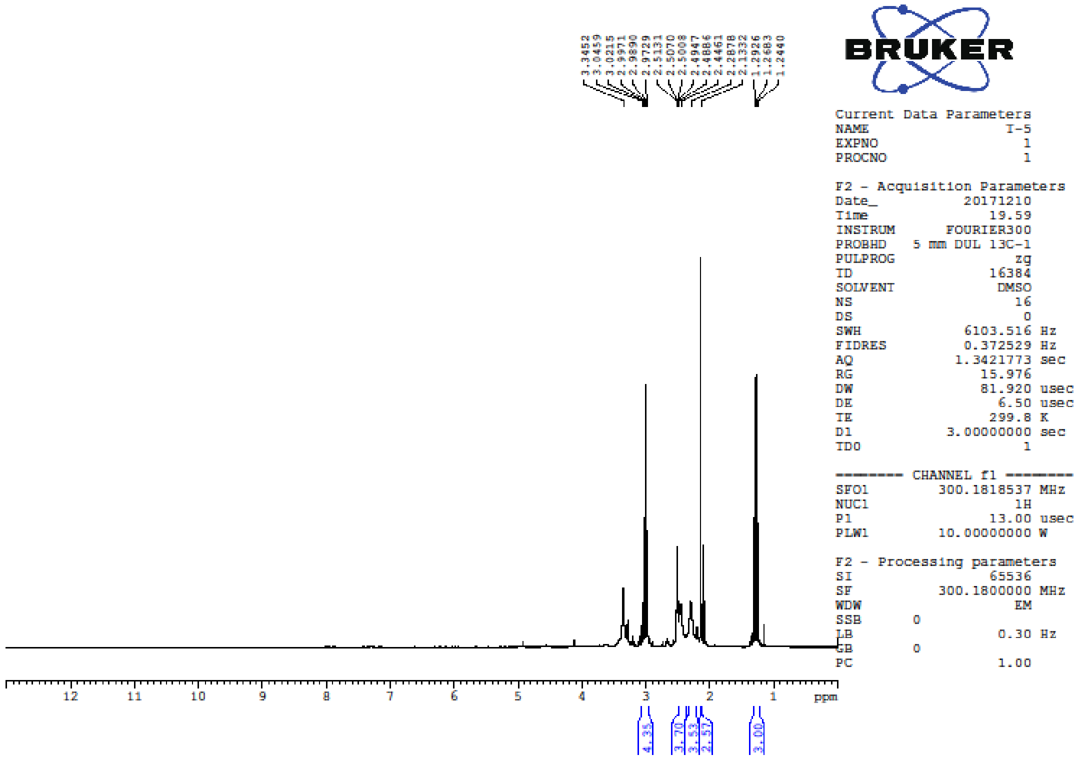

Spectra 19. ^{1}H-NMR spectra of compound **2e**

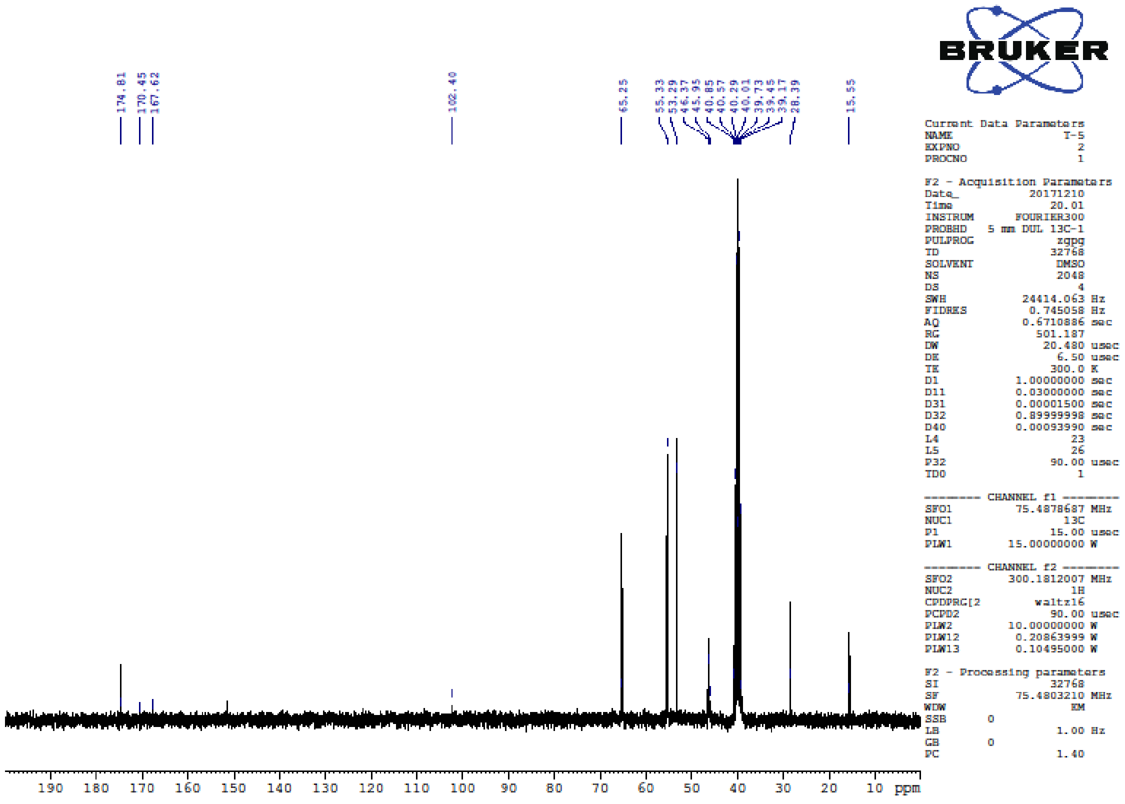

Spectra 20. ^{13}C-NMR spectra of compound **2e**

DOPNALAB

Item	Value
Acquired Date&Time	13.12.2017 09:15:10
Acquired by	System Administrator
Filename	C:\Users\dopnalab\Desktop\derya\T serisi\T-61.ispd
Spectrum name	T-61
Sample name	T-6
Sample ID	
Option	
Comment	
No. of Scans	10
Resolution	4 [cm-1]
Apodization	Happ-Genzel

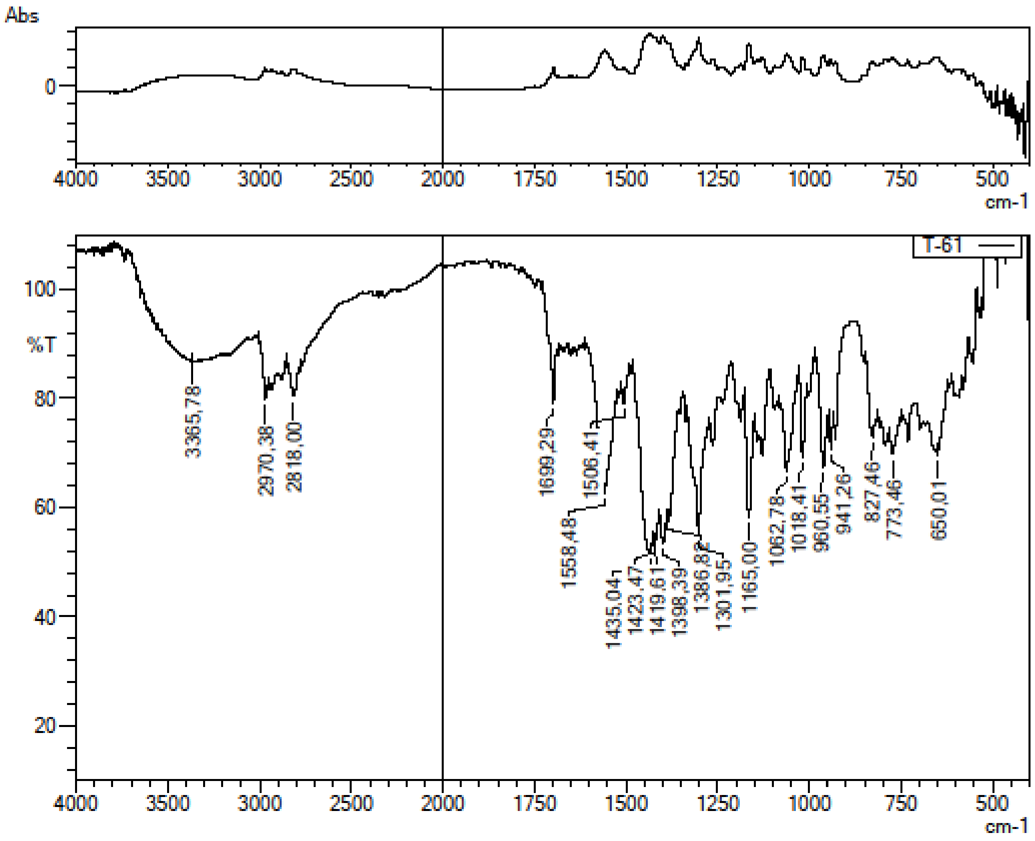

Spectra 21. IR spectra of compound **2f**

LCMSMS ANALYSES REPORT

Sample Name	: T-6		
Sample ID	:		
Data Filename	: T-6_007.lcd		
Method Filename	: genel.lcm		
Batch Filename	:GENEL_batch_tarama.lcb		
Vial #	: 1-21		
Injection Volume	: 1 uL	Sample Type	: Unknown
Date Acquired	: 19.10.2017 14:56:35	Acquired by	:System Administrator
Date Processed	: 19.10.2017 14:58:49	Processed by	:System Administrator

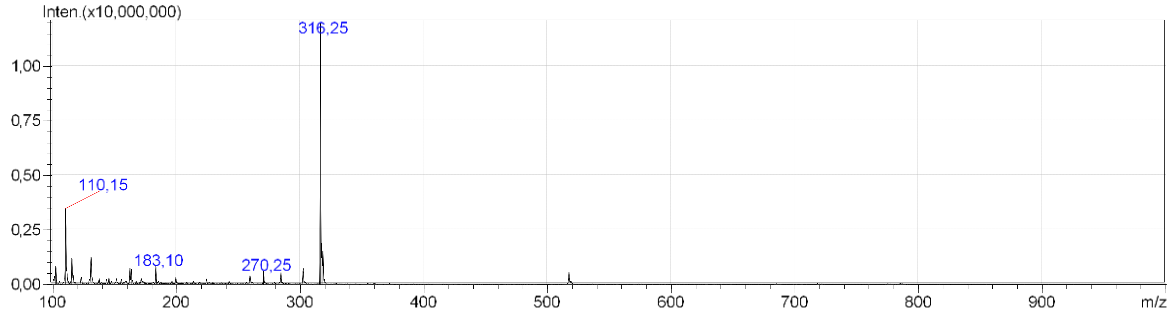

[MS Spectrum]

# of Peaks 12

Raw Spectrum [0,609->0,846],(scan:[37->51])

Background No Background Spectrum

Base Peak m/z 316,25 (Inten : 11.888.338)

Spectra 22. LCMSMS spectra of compound **2f**

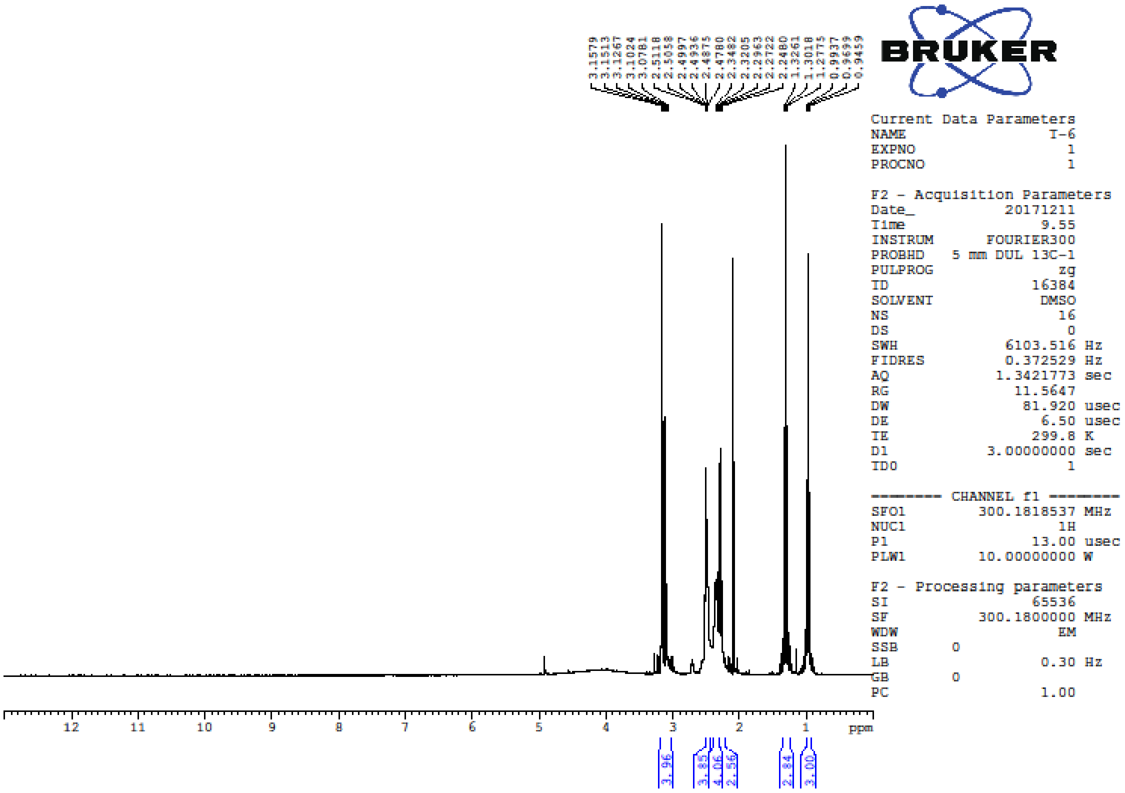

Spectra 23. ^{1}H-NMR spectra of compound **2f**

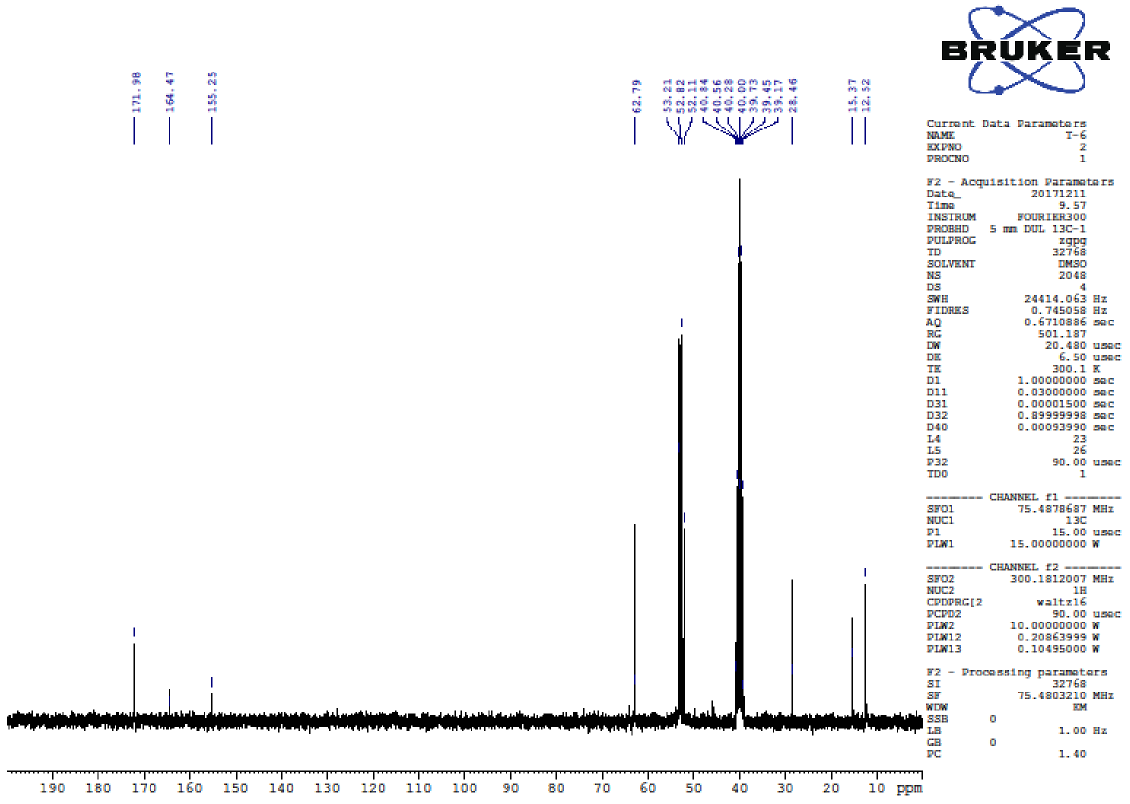

Spectra 24. ^{13}C-NMR spectra of compound **2f**

DOPNALAB

	Item	Value
	Acquired Date&Time	13.12.2017 09:07:10
	Acquired by	System Administrator
	Filename	C:\Users\dopnalab\Desktop\derya\T serisi\T-71.ispd
	Spectrum name	T-71
	Sample name	T-7
	Sample ID	
	Option	
	Comment	
	No. of Scans	10
	Resolution	4 [cm-1]
	Apodization	Happ-Genzel

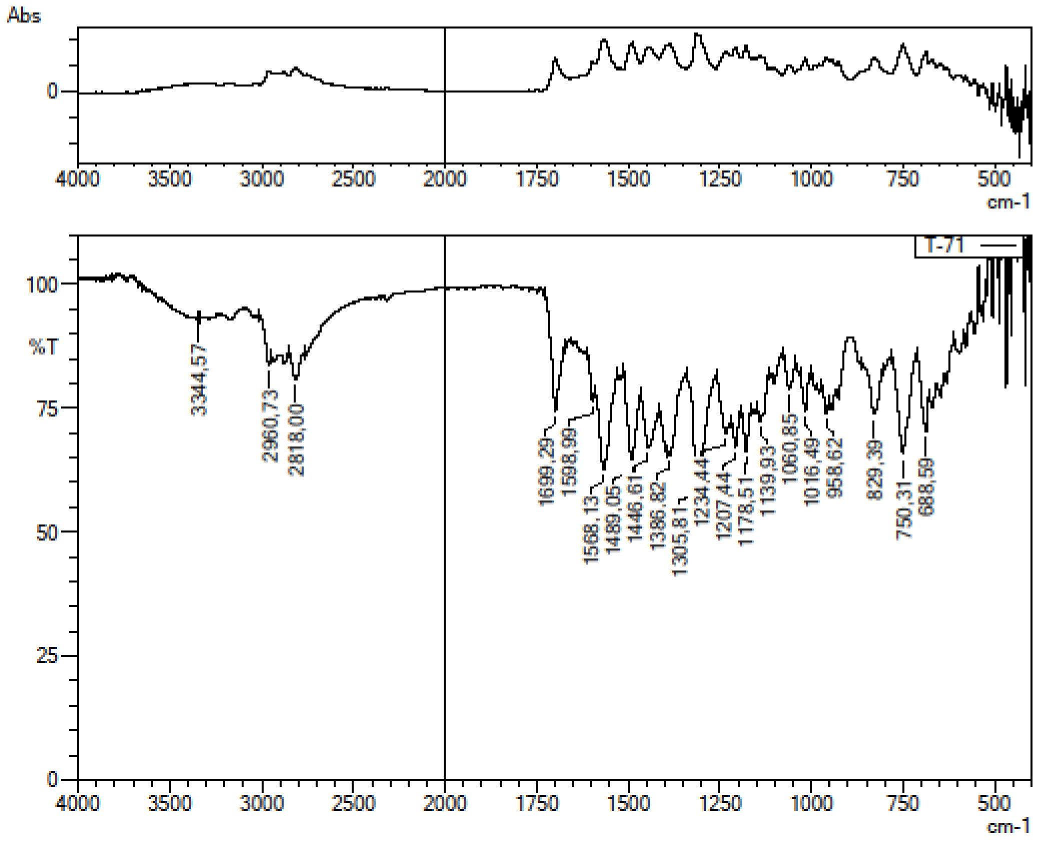

Spectra 25. IR spectra of compound **2g**

LCMSMS ANALYSES REPORT

Sample Name	: T-7		
Sample ID	:		
Data Filename	: T-7_008.lcd		
Method Filename	: genel.lcm		
Batch Filename	:GENEL_batch_tarama.lcb		
Vial #	: 1-22		
Injection Volume	: 1 uL	Sample Type	: Unknown
Date Acquired	: 19.10.2017 14:59:00	Acquired by	:System Administrator
Date Processed	: 19.10.2017 15:01:13	Processed by	:System Administrator

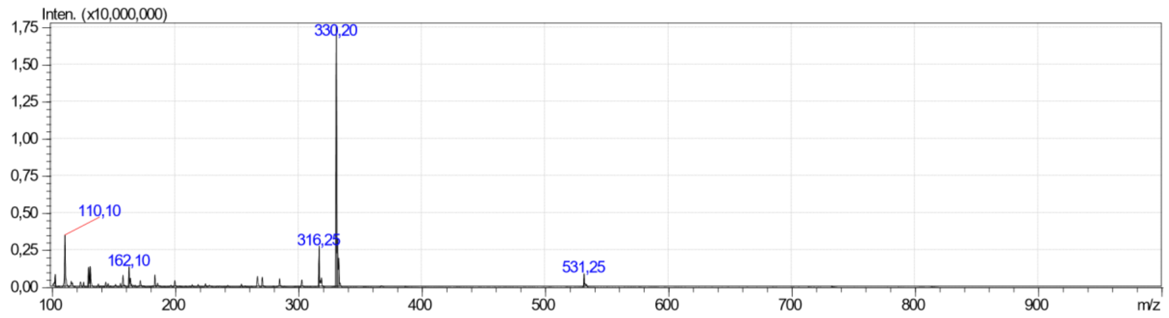

[MS Spectrum]

# of Peaks 10

Raw Spectrum [0,609->0,947],(scan:[37->57])

Background No Background Spectrum

Base Peak m/z 330,20 (Inten : 17.590.258)

Spectra 26. LCMSMS spectra of compound **2g**

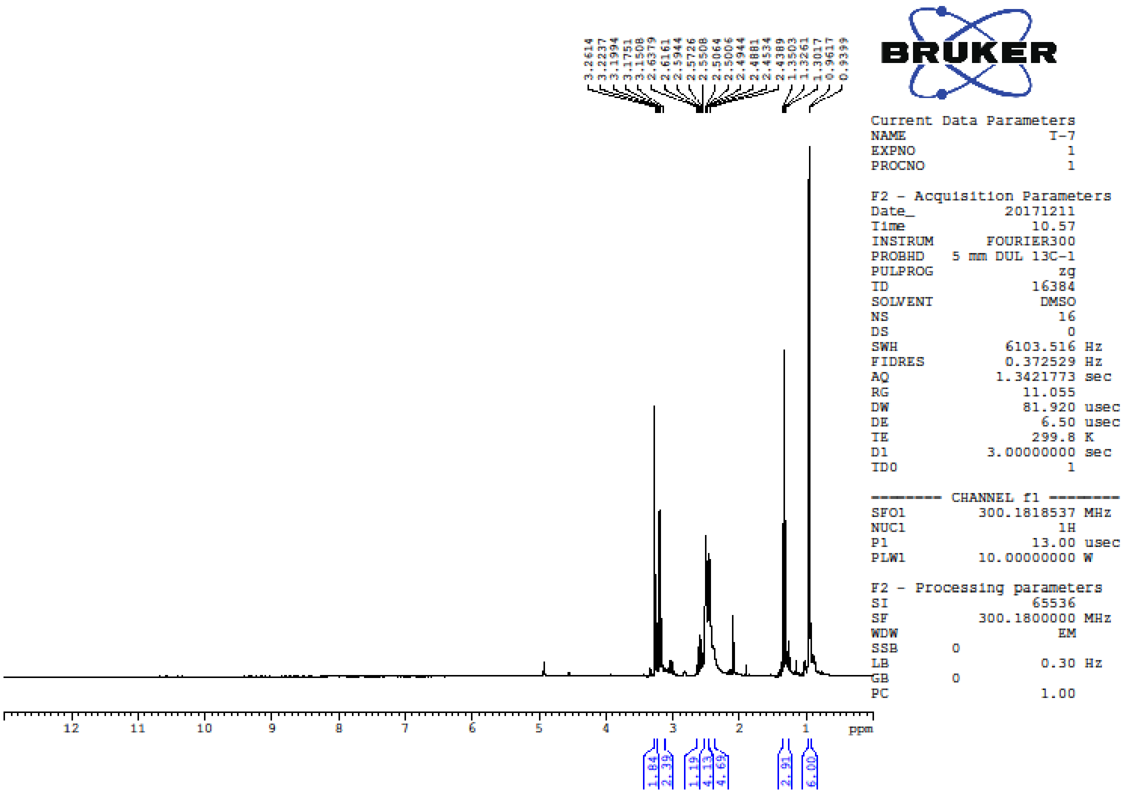

Spectra 27. ^{1}H-NMR spectra of compound **2g**

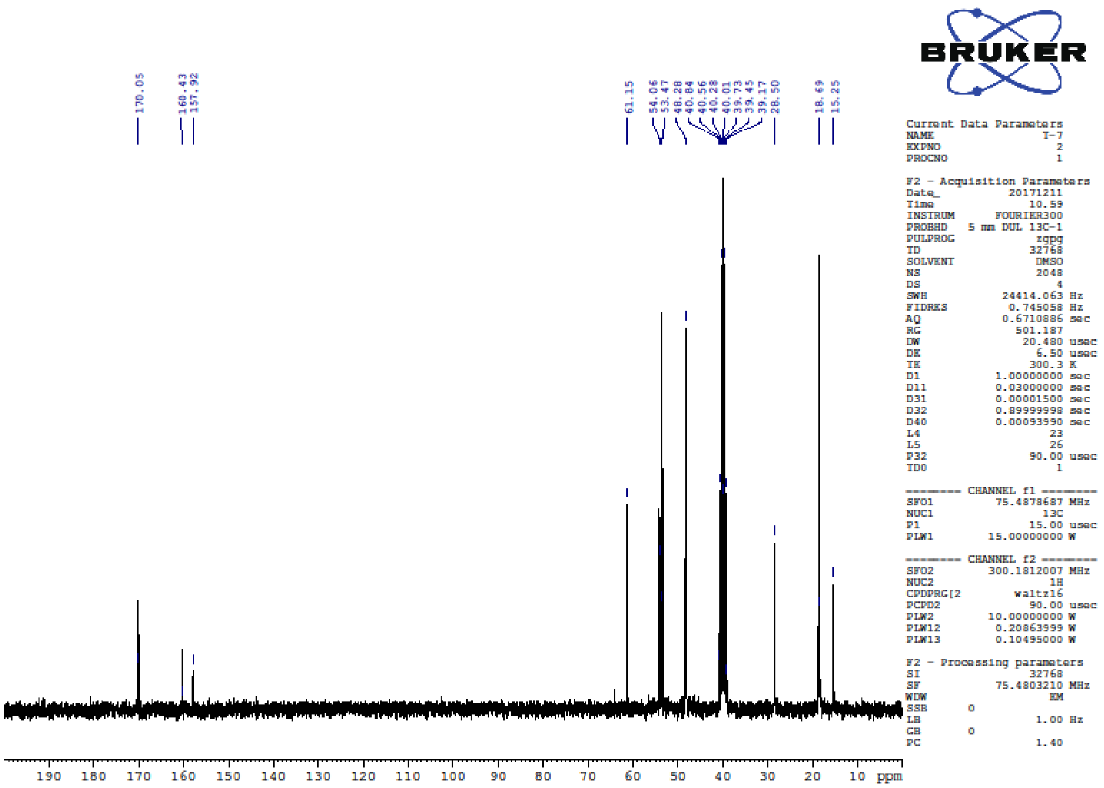

Spectra 28. ^{13}C-NMR spectra of compound **2g**

DOPNALAB

Item	Value
Acquired Date&Time	13.12.2017 09:09:05
Acquired by	System Administrator
Filename	C:\Users\dopnalab\Desktop\derya\T serisi\T-81.ispd
Spectrum name	T-81
Sample name	T-8
Sample ID	
Option	
Comment	
No. of Scans	10
Resolution	4 [cm-1]
Apodization	Happ-Genzel

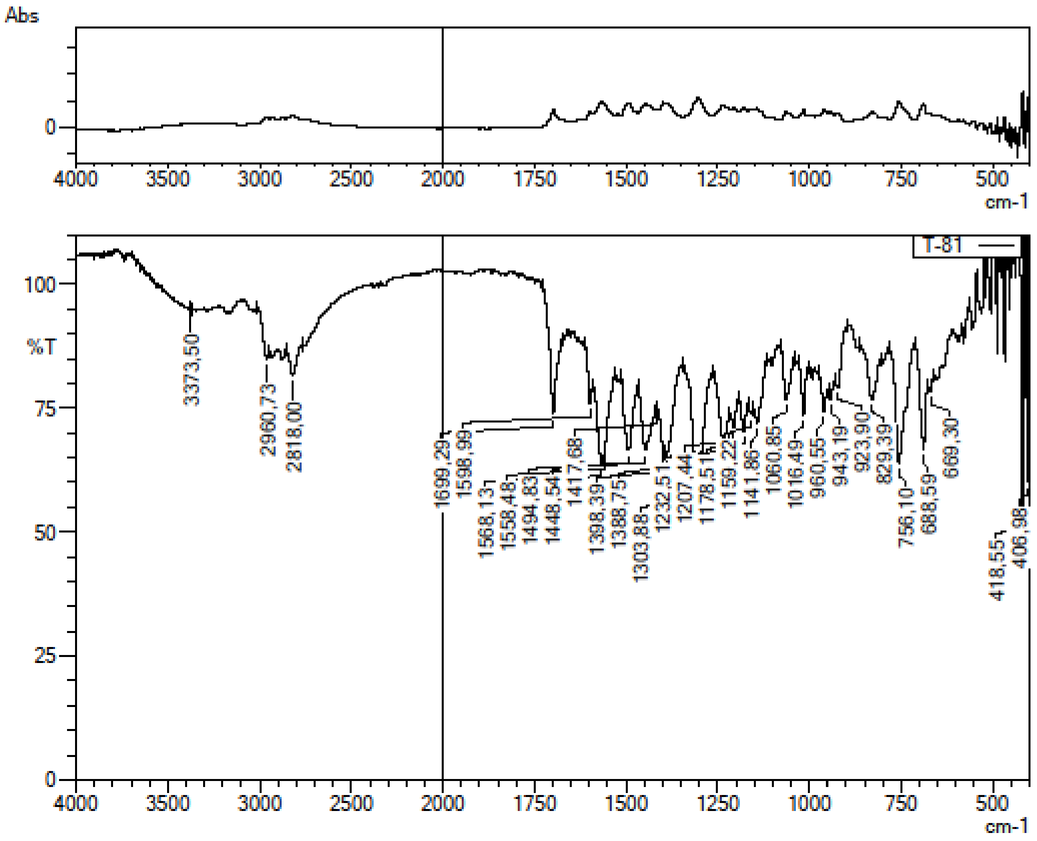

Spectra 29. IR spectra of compound **2h**

LCMSMS ANALYSES REPORT

Sample Name	: T-8		
Sample ID	:		
Data Filename	: T-8_009.lcd		
Method Filename	: genel.lcm		
Batch Filename	:GENEL_batch_tarama.lcb		
Vial #	: 1-23		
Injection Volume	: 1 ul	Sample Type	: Unknown
Date Acquired	: 19.10.2017 15:01:25	Acquired by	:System Administrator
Date Processed	: 19.10.2017 15:03:32	Processed by	:System Administrator

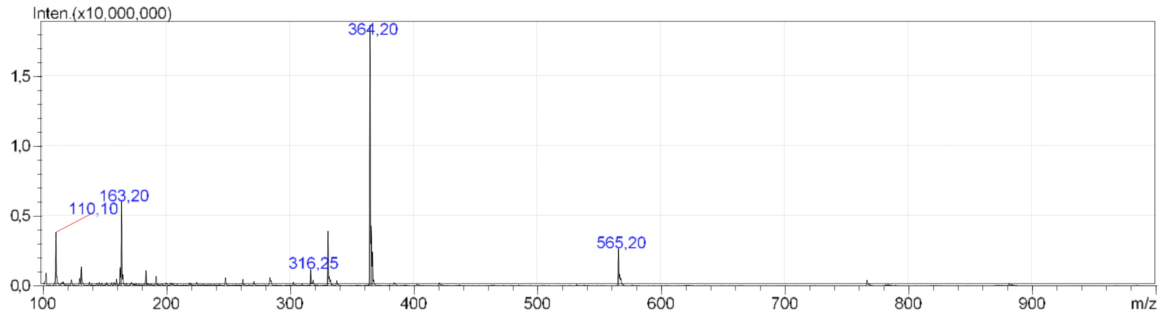

[MS Spectrum]

# of Peaks 11

Raw Spectrum [0,338->0,406],(scan:[21->25])

Background No Background Spectrum

Base Peak m/z 364,20 (Inten : 18.692.104)

Spectra 30. LCMSMSspectra of compound **2h**

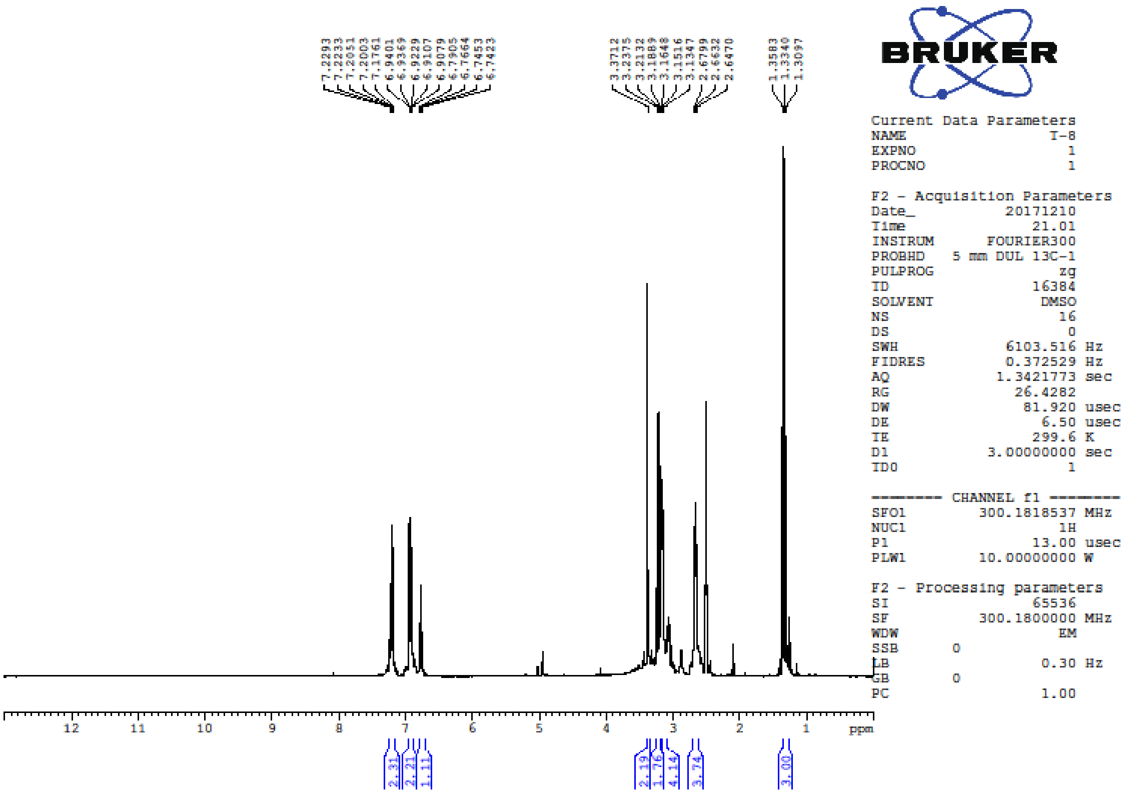

Spectra 31. ^{1}H-NMR spectra of compound **2h**

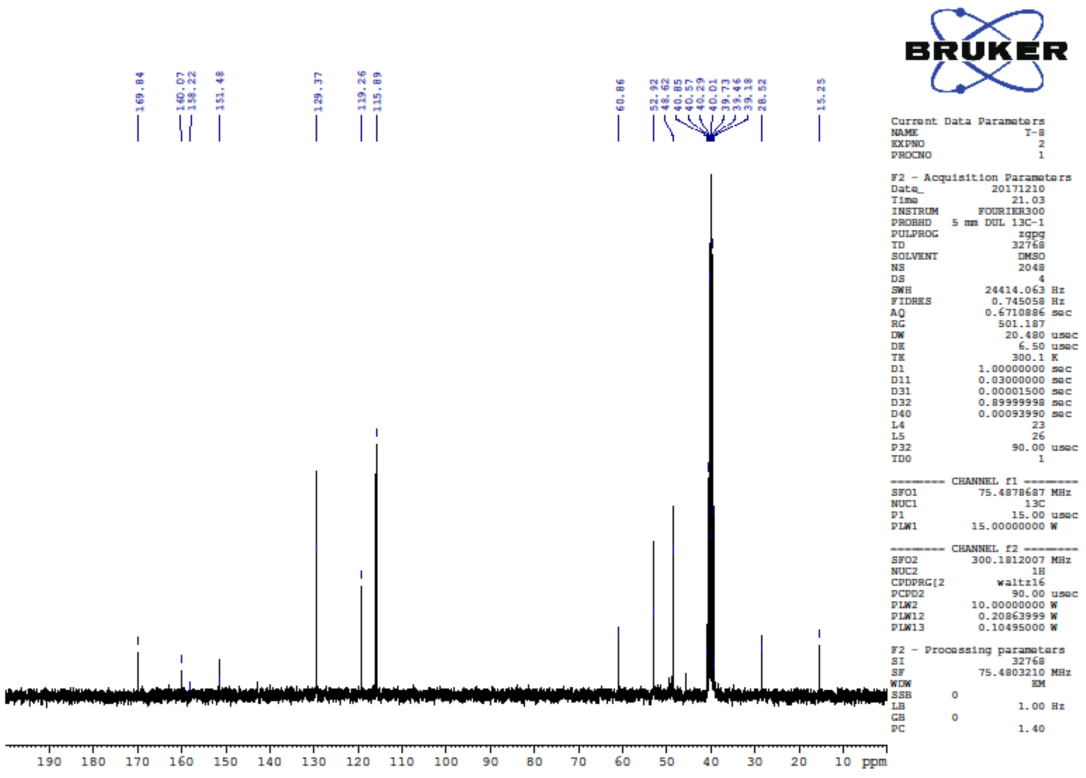

Spectra 32. ^{13}C-NMR spectra of compound **2h**
